# An EPR Strategy for Bio-responsive Fluorescence Guided Surgery with Simulation of the Benefit for Imaging

**DOI:** 10.7150/thno.42702

**Published:** 2020-02-10

**Authors:** Harrison C. Daly, Emer Conroy, Mihai Todor, Dan Wu, William M. Gallagher, Donal F. O'Shea

**Affiliations:** 1 Department of Chemistry, RCSI, 123 St. Stephen's Green, Dublin 2, Ireland.; 2 School of Biomolecular and Biomedical Science, Conway Institute, University College Dublin, Belfield, Dublin 4, Ireland.

**Keywords:** NIR-AZA fluorophore, bio-responsive fluorescence, EPR effect, fluorescence-guided surgery, simulation software

## Abstract

A successful matching of a PEG group size with the EPR effect for an *off-to-on* responsive NIR-fluorophore conjugate has been accomplished which allows two distinct *in vivo* tumor imaging periods, the first being the switch on during the initial tumor uptake via enhanced permeability into the ROI (as background is suppressed) and a second, later, due to enhanced retention within the tumor.

**Methods**: Software simulation (https://mihaitodor.github.io/particle_simulation/index.html), synthetic chemistry, with *in vitro* and *in vivo* imaging have been synergistically employed to identify an optimal PEG conjugate of a bio-responsive NIR-AZA fluorophore for *in vivo* tumor imaging.

**Results**: A bio-responsive NIR-AZA fluorophore conjugated to a 10 kDa PEG group has shown excellent *in vivo* imaging performance with sustained high tumor to background ratios and peak tumor emission within 24 h. Analysis of fluorescence profiles over 7 days has provided evidence for the EPR effect playing a positive role.

**Conclusion**: Preclinical results show that exploiting the EPR effect by utilizing an optimized PEG substituent on a bio-responsive fluorophore may offer a means for intraoperative tumor margin delineation. The *off-to-on* responsive nature of the fluorophore makes tumor imaging achievable without waiting for clearance from normal tissue.

## Introduction

Medical imaging is clinically essential for localization, identification and diagnosis of all cancer types. Today, high definition images of solid tumors are obtained using magnetic resonance, positron emission or computed tomography techniques. Perhaps surprisingly, these images do not play an informative role during the surgical resection 1. The size and complexity of these instruments prohibit their intraoperative use and images taken prior to surgery do not offer further assistance in interpreting the complex boundaries between normal and cancerous tissues and anatomical features encountered in surgical oncology. Tissue and vital structure imaging using light emission from molecular fluorophores offers a viable alternative 2-5. Currently, microscope, open and laparoscopic camera hardware exist which are capable of capturing high quality fluorescence images during surgical procedures without impeding the normal surgical workflow. These devices offer an untapped potential to guide surgical resections in real-time with their inherent ease of use, facilitating continuous imaging during surgical procedures. Historically, fluorescence imaging has its foundations in biomedical research, acting as an essential tool for the investigation of fundamental biological processes. More recently, interest in its *in vivo* use, with near-infrared light (NIR) (*λ* = 700 - 1400 nm), has grown substantially 6. Described as the therapeutic window, this wavelength range is optimal for clinical imaging due to lower tissue auto-fluorescence and attenuation of emitted light.

In spite of the recent advances in instrumentation and software for optical clinical imaging, only a limited number of NIR probes have been developed, with few exhibiting all the criteria needed to be successful as *in vivo* imaging agents 7. Indocyanine green (ICG) is a cyanine based dye and is currently the only FDA and EMA approved NIR-fluorophore 8. Clinical uses include ophthalmic angiography, vascularization assessments during reconstructive and bowel anastomoses surgeries and lymph node mapping 9-12. Due to its non-specificity and very short 4 minute *in vivo* half-life, its use as an agent to demarcate tumor boundaries for surgical resection is restricted to hepatocellular carcinoma of the liver 13, 14. This lack of clinically suitable NIR-emitters has led to the development of new bio-conjugated NIR-fluorophores with enhanced affinity for cancers over normal tissue. Bio-conjugating groups used include antibodies bevacizumab, cetuximab and carcinoembryonic antigen (CEA) 15-18, peptides 19 and small molecules such as folic acid 20. Recent clinical trials for visualizing breast, colorectal, head / neck and brain cancers have been conducted utilizing NIR-fluorophore labelled bevacizumab, CEA and cetuximab antibodies respectively 15-18. Very encouraging results have been obtained in each trial, but despite using expensive cancer specific antibody technologies, tumor images with sufficient contrast could only be acquired between two and seven days post administration. The prolonged waiting period to achieve contrast between cancerous and normal tissues is due to the very long biological half-lives of antibody labelled agents in comparison to use of the low molecular fluorophore alone. This time limitation occurs in spite of the antibody-endowed tumor specificity, as an initial broad distribution of fluorophore will still occur and the high molecular weight antibody will remain in the vasculature for days. The time between administration and imaging depends on several parameters such as rates of accumulation and clearance from both the tumor and surrounding tissues via metabolic and excretion pathways. For targeting to take place, the antibody must first accumulate immediately adjacent to the tumor for receptor binding to occur. The bulk of the labeled fluorophore will not reach the specific tumor site for binding and will remain as prolonged background fluorescence. The time taken for the clearance of this background interference is an often-overlooked factor in the development of targeted contrast agents. This time delay adds significant uncertainty to their practical use and raises doubts as to whether the antibody is of overall benefit despite its active targeting of the tumor. These results prompted us to explore the potential of pegylated NIR-fluorophores, as they may offer an inexpensive passive cancer targeting delivery system by exploiting the enhanced permeability and retention (EPR) effect 21.

### Rationale for Passive Targeting Strategy

**Figure 1:**
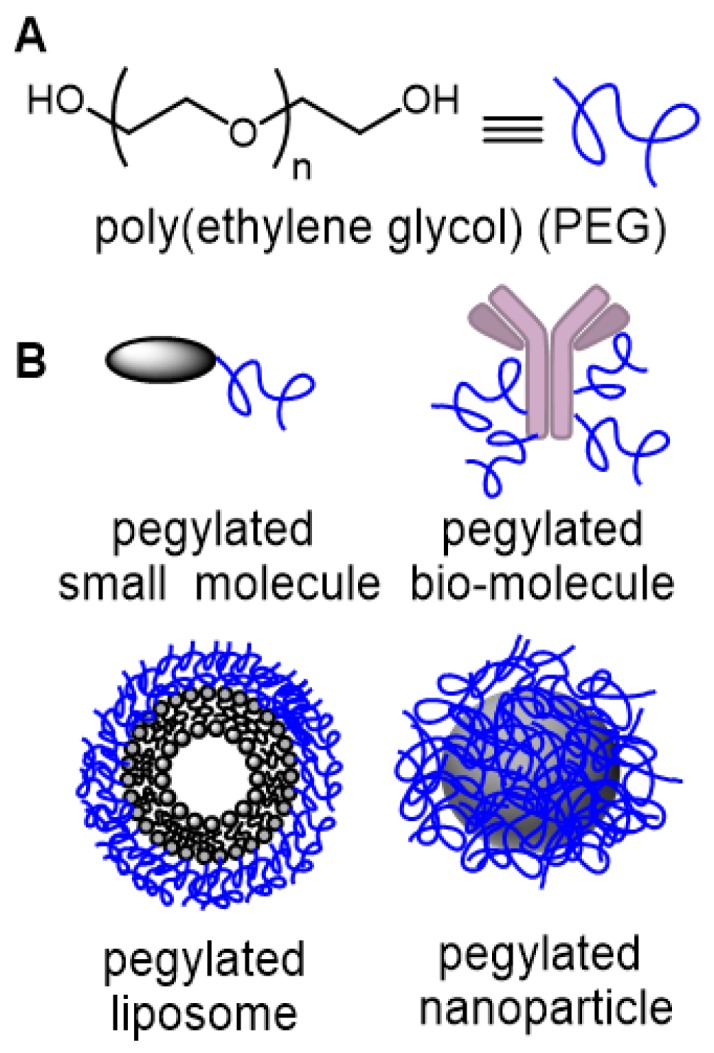
(A) Structure of poly(ethylene glycol) (PEG). (B) Common drug delivery applications of PEG groups as covalent conjugates of small molecules, large bio-molecules, liposomes and nanoparticles.

Angiogenesis in the cancer disease state is rapid, leading to blood vessels with defective architecture, characterized by wide inter-endothelial junctions, large number of fenestrae and transendothelial channels and a discontinuous or absent basement membrane 22. This causes a significantly higher permeability of the endothelial barrier of tumor vasculature compared with normal tissue, resulting in not only the accumulation of macromolecules in the tumor interstitium but also importantly their retention due to missing or decreased lymphatic drainage. As this abnormal blood flow into and lymphatic drainage from tumors is exploited for drug delivery, we reasoned that it could also be exploited for imaging. The EPR effect is a unique, molecular weight and size dependent phenomenon, in which large molecules or particles tend to accumulate over time in solid tumors more than normal tissues, due to these anatomical defects 23. The covalent attachment of polyethylene glycols (PEG) to a drug molecule or delivery vehicle (Figure 1) is the most successful strategy to exploit the EPR effect for passive tumor targeting 24. First reported in the late 1970s, and with the subsequent discovery of the EPR effect, pegylation strategies have been widely used in the pharmaceutical industry to improve the clinical performance of several drug candidates 25. With respect to drug delivery, pegylation designates the covalent attachment of one (or more) PEG chains either to a low molecular weight drug, a large bio-molecule or to the delivery vector for the drug molecule such as liposomes, or nanoparticles (Figure 1). PEGs are non-toxic, non-immunogenic, non-antigenic, FDA approved polymers for human oral, intravenous and dermal pharmaceutical applications and are cleared through renal and hepatic pathways 26.

Fast renal clearance can be avoided by conjugating small molecules with PEGs, whereas macromolecular conjugation with low molecular weight PEG can mask cationic charges, reduce enzymatic degradation and avoid opsonization and subsequent elimination by the reticuloendothelial system (RES) 27. Consequently, pegylation gives therapeutics a number of favorable properties such as increased aqueous solubility, prolonged residence time in the vascular system (increased half-life), reduced interaction with enzymes and antibodies, decreased immunogenicity and passive accumulation in tumors.

**Figure 2:**
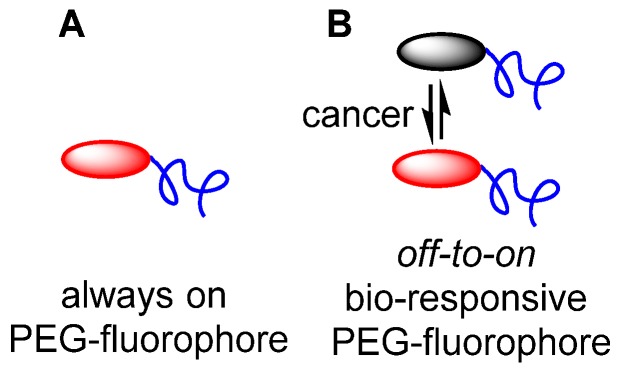
(A) Pegylated *always-on* fluorophore. (B) Pegylated bio-responsive *off-to-on* pegylated fluorophore.

To date, the majority of clinically approved pegylated drugs are proteins, antibody fragments or oligonucleotides 28, 29. Translation of small molecule chemotherapeutics is not as advanced, with PEG variants of camptothecin, doxorubicin, paclitaxel, cisplatin, wortmannin, gemcitabine and methotrexate reported in the research literature, and several entering clinical trials 26. Clinical challenges facing pegylated small drug molecules are the need for site specific release of the drug from the PEG group and the relatively low drug loading with respect to the amount of PEG 30. Neither of these are limiting factors for pegylated fluorophores as release is not required and high loading is unnecessary, as PEG groups are known to increase fluorescence quantum yields in aqueous environments (Figure 2A).

As the primary surgical goal is to map the extent of tumor margins as a guide for resection, it is unnecessary to have the fluorophore penetrate deep into the tumor, which is consistent with the EPR effect being restricted to the outer tumor boundary. Yet, consideration must also be given to the issue that surgical fluorescence image capture relies on having sufficient contrast between tumor and surrounding tissues at precisely the moment that it is needed i.e. during the operation. An *always-on* fluorescent probe that initially has perfused through all tissues necessitates waiting for clearance from non-cancerous tissues and retention in the tumor. Exploitation of the EPR effect for fluorophores is plausible but similar to an antibody conjugate, would require a prolonged time between administration and imaging. An alternative approach would be to utilize a bio-responsive fluorophore, which has a trigger component to switch fluorescence from *off-to-on* (Figure 2B) 31. If the off state was favored in the vasculature, this could suppress background fluorescence while allowing emission to first switch on once accumulation occurs within the cancerous growth.

**Figure 3:**
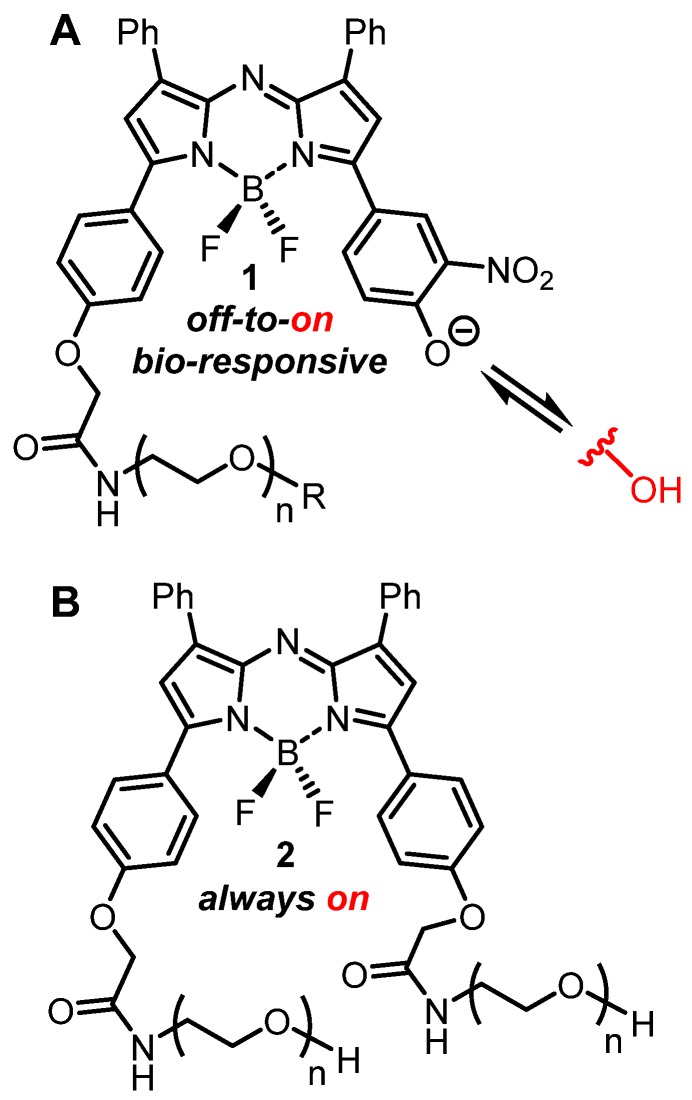
(A) General structure of bio-responsive *off-to-on* NIR-AZA fluorophores **1** used in this study. (B) General structure of *always-on* pegylated NIR-AZA fluorophore **2**.

In order to test the potential of an EPR mediated imaging approach, both *always-on* and bio-responsive fluorophores attached to PEG groups of varying sizes have been studied. The NIR-AZA class of fluorophore was chosen as it has excellent photophysical properties such as NIR-wavelengths and high photostability, and is directly translatable between *in vitro* and *in vivo* imaging 32-36. The bio-responsive derivatives **1** have a phenolate/phenol switch which regulates the emission such that in extracellular media with pH of 7.2 the phenolate exists and the emission is off, whereas in lower intracellular pH regions (late endosomes and lysosomes) the phenol predominates as an emissive species (Figure 3A) 36. Pegylated *always-on* derivate **2** structurally has an additional PEG unit instead of the switching component and was also included in the study (Figure 3B) 39.

Each fluorescent construct was imaged *in vitro* and *in vivo* using MDA-MB 231 human breast cancer models to gauge the impact on tumor imaging of PEG size and bio-responsive versus *always-on* emissions. In addition to gaining insight into the potential for fluorescence-guided surgery (FGS), it was anticipated that the bio-responsive derivatives may also illustrate the key uptake and retention properties attributable to the EPR effect. To illustrate this, a software simulation model was developed to show the imaging effect of key parameters such as rates of uptake and clearances from normal and cancerous tissues, effect of EPR on imaging contrast and the benefits of utilizing fluorophores that switch from *off-to-on* upon reaching their target.

## Materials and Methods

### Imaging simulation

The JavaScript framework Three.js https://threejs.org/ was used to build the central animation consisting of the chamber (FOV), inputs and output tubes, EPR zone, target region of interest (ROI) and fluorescent agent. Three.js makes it possible to author complex 3D WebGL-based https://www.khronos.org/webgl/ animations without the effort required for a traditional standalone computer application. It provides a high-level application programming interface (API) which lets the user write code to create various 3D shapes (spheres, boxes, tubes), specify their properties (color, dimensions, position) and how they will move in the scene. The aspect and position of all Three.js shapes can be modified programmatically in real time using simple linear algebra transformations through various Three.js API calls. Additionally, as needed, Three.js contains functionality to enable users to interact with the shapes or change the view in real time using input devices such as a mouse.

The data display chart was built using the JavaScript framework C3.js https://c3js.org/. C3.js provides a high level API for defining various chart properties and allows users to insert new data points programmatically while the animation is running. It is built on top of a library called D3.js https://d3js.org/ that uses scalable vector graphics (SVG) browser technology for rendering and animating charts. The side input menu, which lets users adjust and select various options for controlling the aspect of the animation and chart, was built using the dat.GUI.js https://github.com/dataarts/dat.gui JavaScript framework. This framework provides APIs for creating controls and adding JavaScript callbacks which alter the aspect and behavior of the animation and chart based on user input. It also enables the saving of configurations as a custom profile and exporting of results data shown in the plots as Excel CSV files.

The simulation can be accessed at https://mihaitodor.github.io/particle_simulation/index.html. The software code is available as open source on GitHub https://github.com/mihaitodor/particle_simulation and permits a user to modify and save revised versions. The central animation components (FOV, EPR zone, ROI and tubes) are positioned statically within the Three.js scene. The user can use the mouse and keyboard to interact with this component ensemble and rotate it around by pressing the left click button, zoom in and zoom out by using the scroll wheel and, finally, drag it around by pressing the Ctrl key in combination with the left click button.

At the start of the animation, all the fluorescent agent particles are invisible and they are positioned at the injection site. Each of them is assigned a random velocity vector pointing towards the interior of the FOV. After the initialization routine finishes, the particle animation starts immediately and the particles are released in batches of 100 in random directions every 5 frames. Each frame, every visible particle's position is advanced along the particle's rectilinear trajectory by adding the particle's velocity vector to its current position. Particles do not interact with each other. When a particle interacts with a surface, the following cases are possible:

if a particle collides with one of the FOV's boundary walls, it is reflected back;

if the EPR zone is active and a particle inside it collides with its boundary surface, the particle is either reflected back or it can escape back into the FOV;

if a particle is inside the ROI and it collides with its boundary surface, the particle is reflected back.

When the EPR zone is enabled, the trajectories of particles crossing it are influenced by a gravity-like effect, being directed towards the ROI. Due to this effect, most particles do not escape back into the FOV after they entered the EPR zone. The velocity of particles in the EPR zone and in the ROI differs by a constant viscosity factor from the velocity in the FOV. The viscosity in the EPR zone is 0.9, thus the velocities of particles inside it are 0.1 x FOV velocities. The viscosity in the ROI is 0.155, so the velocities of particles inside it are 0.845 x FOV velocities. Both the FOV and ROI have a configurable initial delay and clearance rate before fluorophore clearance commences. The clearance rates dictate how many random particles are selected from the FOV (every 20 frames) and how many are selected from the ROI (every 40 frames). The selected particles are positioned at the clearance site. For the purpose of clearance, when the EPR zone is enabled, particles inside it are considered as being in the FOV. The % FOV / ROI distributions over time chart is updated each 30 frames. Depending on the performance of the system on which the simulation is running, the maximum number of frames per second is 60 (as can be observed in the FPS box in the top left corner), so the chart will be updated at most twice per second.

### Synthetic chemistry

All reactions involving air-sensitive reagents were performed under nitrogen in oven-dried glassware using syringe-septum cap techniques. All solvents were purified and degassed before use. Chromatographic separation was carried out under pressure on Merck silica gel 60 using flash-column techniques. Reactions were monitored by thin-layer chromatography (TLC) carried out on 0.25 mm silica gel coated aluminum plates (60 Merck F254) using UV light (254 nm) as visualizing agent. Unless specified, all reagents were used as received without further purifications. ^1^H NMR and ^13^C NMR spectra were recorded at rt at 400 MHz or 600 MHz and 100 MHz respectively and calibrated using residual non-deuterated solvent as an internal reference. ESI mass spectra were acquired using Advion Expression Mass Spectrometer in positive and negative modes as required. Advion Data Express v5.1 software were used to carry out the analysis. ESI (HRMS) mass spectra were acquired using a microTOF-Q spectrometer interfaced to a Dionex UltiMate 3000 LC in positive and negative modes as required. MicroTof control 3.2 and HyStar 3.2 software were used to carry out the analysis. The desalting purification was completed via size exclusion chromatography Sephadex G-25 (30 × 300 mm) and analyzed by reverse phase chromatography on a HPLC (Shimadzu) equipped with analytical (YMC-triart phenyl, 4.6 × 150 mm I.D. S-5 μm, 12 nm) columns, eluent with acetonitrile / water. Combined pure fractions were dried by lyophilization. All absorbance spectra were recorded with a Varian Cary 50 scan UV-visible spectrophotometer and fluorescence spectra were recorded with a Varian Cary eclipse fluorescence spectrophotometer. Data was normalized in SigmaPlot 8, pKa values were generated from plots of pH values on the x-axis and integrated fluorescent intensity values on the y-axis using the dynamic curve fit function.

#### Preparation of 2-(4-(5,5-difluoro-7-(4-hydroxy-3-nitrophenyl)-1,9-diphenyl-5*H*-4λ4,5λ4-dipyrrolo[1,2-c:2',1'-f][1,3,5,2]triazaborinin-3-yl)phenoxy)-*N*(polyethylene glycolyl)acetamide, **1a** 38

To a mixture of **3** (6.4 mg, 0.0088 mmol) and *O*-(2-aminoethyl)polyethylene glycol 5000 (40 mg, 0.008 mmol) in a N_2_ flushed 1.5 mL round bottomed flask, anhydrous DMSO (1 mL) was added. The reaction mixture was sonicated well and set to stir at rt for 6 h under a N_2_ atmosphere. The reaction was diluted with 10 volumes of HPLC water and lyophilized before being partitioned between DCM (20 mL) and 1M Na_2_CO_3_ (20 mL). The aqueous phase was extracted with DCM (2 × 20 mL). The organic layers were combined, washed with 0.5 M HCL (20 mL), brine (20 mL), dried over anhydrous Na_2_SO_4_, filtered and evaporated to dryness *in vacuo*. The residue was dissolved in HPLC grade water (8 mL) and the dark solution was passed through a Sep Pak C18 reverse phase cartridge and lyophilized. The resulting material was dissolved in CH_3_CN:H_2_O (60:40), acidified with 0.5 M HCl, filtered through a PTFE 0.45 μM syringe filter and the resulting dark green solution was purified by reverse phase semi prep chromatography (YMC-triart phenyl, 10 × 150 mm I.D., eluent CH_3_CN:H_2_O 60:40). Fractions containing pure product were pooled, concentrated *in vacuo* and lyophilized to give the product **1a** as a dark green solid (26 mg, 60%). ^1^H NMR (400 MHz, DMSO-d_6_) δ: 8.71 (d, *J* = 2.1 Hz, 1H), 8.28 (dd, *J* = 9.0, 2.3 Hz, 1H), 8.26 - 8.14 (m, 7H), 7.73 (s, 1H), 7.65 (s, 1H), 7.60 - 7.45 (m, 7H), 7.28 (d, *J* = 9.0 Hz, 1H), 7.16 (d, *J* = 9.0 Hz, 2H), 4.67 (s, 2H), 3.50 (s, 656 H) ppm. MALDI-TOF analysis: distribution maximum centered at 5514.15 Da.

#### Preparation of 2-(4-(5,5-difluoro-7-(4-hydroxy-3-nitrophenyl)-1,9-diphenyl-5H-4λ4,5λ4-dipyrrolo[1,2-c:2',1'-f][1,3,5,2]triazaborinin-3-yl)phenoxy)-*N*(polyethylene glycolyl)acetamide, **1b**

To a mixture of **3** (3.5 mg, 0.0048 mmol) and *O*-(2-aminoethyl)polyethylene glycol 10,000 (40 mg, 0.004 mmol) in a N_2_ flushed round bottomed flask, anhydrous DMSO (2 mL) was added. The reaction mixture was sonicated well and set to stir at r.t. for 6 h under a N_2_ atmosphere. The reaction was diluted with 10 volumes of HPLC water and lyophilized before being partitioned between DCM (20 mL) and 1M Na_2_CO_3_ (20 mL). The aqueous phase was extracted with DCM (2×20 mL). The organic layers were combined, washed with 0.5 M HCl (20 mL), brine (20 mL), dried over anhydrous Na_2_SO_4_, filtered and evaporated to dryness *in vacuo*. The residue was dissolved in HPLC grade water (8 mL) and the dark solution was passed through a Sep Pak C18 reverse phase cartridge and lyophilized. The resulting material was dissolved in CH_3_CN:H_2_O (60:40), acidified with 0.5 M HCl, filtered through a PTFE 0.45 μM syringe filter and the dark green solution was purified by reverse phase semi prep chromatography (YMC-triart phenyl, 10 × 150 mm I.D., eluent CH_3_CN:H_2_O 60:40). Fractions containing product were pooled, concentrated *in vacuo* and then lyophilized to give the product **1b** as a dark green solid (29 mg, 70%). ^1^H NMR (400 MHz, DMSO-d_6_) δ: 8.74 (s, 1H), 8.28 (dd, *J* = 9.0, 2.2 Hz, 1H), 8.24 - 8.14 (m, 7H), 7.68 (d, *J* = 11.0 Hz, 2H), 7.59 - 7.51 (m, 4H), 7.52 - 7.45 (m, 2H), 4.66 (s, 2H), 3.50 (s, 976 H) ppm. MALDI-TOF analysis: distribution maximum centered at 9862.03 Da.

#### Preparation of 2-(4-(5,5-difluoro-7-(4-hydroxy-3-nitrophenyl)-1,9-diphenyl-5H-5λ4,6λ4-dipyrrolo[1,2-c:2',1'-f][1,3,5,2]triazaborinin-3-yl)phenoxy)-N-(2-methoxyethylene glycolyl)acetamide, **1c**

Anhydrous DMSO (2 mL) was added to a mixture of **3** (2.4 mg, 0.0032 mmol) and *O*-(2-aminoethyl)-*O*′-methylpolyethylene glycol 20,000 (50 mg, 0.0025 mmol) in a N_2_ flushed round bottomed flask. The reaction mixture was sonicated well and set to stir at rt for 6 h under a N_2_ atmosphere. The reaction was diluted with 10 volumes of HPLC water and lyophilized before being partitioned between DCM (20 mL) and 1M Na_2_CO_3_ (20 mL). The aqueous phase was extracted with DCM (2×20 mL). The organic layers were combined, washed with 0.5 M HCl (20 mL), brine (20 mL) and evaporated to dryness *in vacuo*. The residue was dissolved in HPLC grade water (8 mL) and the dark solution was passed through a Sep Pak C18 reverse phase cartridge and lyophilized. The isolated material was dissolved in CH_3_CN:H_2_O (60:40), acidified with 0.5 M HCl, filtered through a PTFE 0.45μM syringe filter and the dark green solution was purified by reverse phase semi prep chromatography (YMC-triart phenyl, 10 × 150 mmI.D., eluent CH_3_CN:H_2_O 60:40). Fractions containing product were pooled, concentrated *in vacuo* and then lyophilized to give the product **1c** as a dark green solid (29.4 mg, 46%). Attempts to obtain MALDI-TOF analysis of the 20,000 Da amino-PEG reagent or **1c** were unsuccessful due to poor sample desorption/ionization. ^1^H NMR (600 MHz, DMSO-d_6_) δ: ^1^H NMR (600 MHz, DMSO-d_6_) δ: 8.97(s, 1H), 8.33 - 7.88 (m, 8H), 7.57-7.43 (m, 6H), 7.24 - 6.94 (m, 4H), 4.57 (s, 2H), 3.58-3.46(m, 1955H) ppm.

#### Preparation of **2a** 39

A mixture of 2,2'-(((5,5-difluoro-1,9-diphenyl-5*H*-4,5-dipyrrolo[1,2-*c*:2',1'-*f*][1,3,5,2]triazaborinine-3,7-diyl)bis(4,1-phenylene))bis(oxy))diacetic acid (20 mg, 0.0238 mmol) and *O*-(2-aminoethyl) polyethylene glycol 5000 (CAS 32130-27-1) (232 mg, 0.0464 mmol) was dissolved in anhydrous DMSO (2 mL) and stirred at rt for 1 h under a N_2_ atmosphere. The solvent was removed by lyophilization and the crude product partitioned between CH_2_Cl_2_ (20 mL) and H_2_O (20 mL). The aqueous phase was extracted with CH_2_Cl_2_ (2 × 20 mL). The organic layers were combined, washed with aqueous HCl (20 mL, pH 5), water (20 mL), brine (20 mL), dried over anhydrous Na_2_SO_4_, filtered and evaporated to dryness. The residue was dissolved in HPLC grade water (10 mL), passed through a Sep Pak C18 reverse-phase column, and lyophilized. The product **2a** was obtained as a dark green solid (217 mg, 83%), m.p. 62-64 °C. ^1^H NMR (400 MHz, CDCl_3_): δ 8.09-8.03 (m, 8H), 7.48-7.43 (m, 6H), 7.09-6.98 (m, 6H), 4.59 (s, 4H), 3.93-3.80 (m, 8H), 3.79-3.50 (m, 988H), 3.48-3.45 (m, 8H) ppm.

### Dynamic light scattering (DLS) measuring

Particle size and polydispersity index (Pdi) were measured using a Zetasizer NanoZS (Malvern Instrument, Malvern, UK) with a 633 nm wavelength He-Ne laser and scattering angle of 173°. Samples were prepared in PBS and the solution passed through a 0.22 μm filter (Merck Millipore) directly into a disposable cuvette. Measurements were made in triplicate at 20 °C and 37 °C. Size and Pdi of PEG conjugates were measured with 300 s equilibration time. All measurements were performed in triplicate with data analyzed using Zetasizer Nano software (version 7.13).

### Photophysical response to pH

Separately, compounds **1a**-**c** were accurately weighed (mg scale, 4 decimal places) and dissolved in PBS 1x (500 μL). The stock solution was diluted to the concentration of 5 μM with PBS 1x containing TX-100 0.34mM (8 mL total volume). The pH of the solution under stirring was adjusted with diluted HCl or NaOH (0.1/0.5 M) to obtain a range from 8 to 2 at intervals of 0.5, each of which was recorded and the solution analyzed by UV-Vis absorption and fluorescence emission. Excitation = 630 nm, emission range = 650 - 900 nm; slit widths: 5/5.

### Cell culture and live-cell imaging

MDA-MB 231 cells obtained from Caliper Life Sciences. MDA-MB 231 human breast cancer cells were seeded on to an eight well chamber slide (Ibidi) at a density of 1 × 10^4^ cells per well 24 h before imaging. Cells were cultured in Dulbeccos Modified Eagles Media supplemented (DMEM) with 10% fetal bovine serum (FBS), 1% L-Glutamine, and penicillin-streptomycin (1000 U/mL), and incubated at 37 °C and 5% CO_2_. The slide was place on the microscope stage surrounded by an incubator to maintain the temperature at 37 °C and CO_2_ at 5%. DIC imaging was used to choose a field of view and focus on a group of cells. Fluorescence and DIC images were acquired on an Olympus IX73 epi-fluorescent microscope fitted with an Andor iXon Ultra 888 EMCCD and controlled by MetaMorph (v7.8). Fluorescence illumination was provided by a Lumencor Spectra X light engine containing a solid-state light source. NIR: excitation filter = 640 (14) nm, emission filter = 705 (72) nm. Images were acquired using a 60×/1.42 oil PlanApo objective (Olympus). Image processing was completed by using software ImageJ 1.52n (National Institutes of Health, USA).

### *In vivo* mouse imaging

All *in vivo* experiments were conducted in University College Dublin (UCD), Ireland in compliance with EU Directive 2010/63 EU. Experiments were approved by the Health Products Regulatory Authority (Authorization number: AE18982/P039) and UCDs Animal Research Ethics Committee. All *in vivo* work was carried out in the biomedical facility, UCD. MDA-MB-231, a human breast adenocarcinoma cell line, was obtained from Caliper Life Sciences. Cells were maintained as a mono-layer culture in Minimum Essential Medium containing 10% (v/v) fetal bovine serum and supplemented with 1% (v/v) L-glutamine, 50 U/mL penicillin, 50 μL/mL streptomycin, 1% (v/v) sodium pyruvate and 1% (v/v) non-essential amino acids. All cells were maintained in 5% CO_2_ (v/v) and 21% O_2_ (v/v) at 37 °C. Balb/C nu/nu mice (Harlan) were housed in individually ventilated cages in temperature and humidity-controlled rooms with a 12 h light dark cycle. 2-5 Million MDA-MB 231 cells in 100 μL of a DPBS:Matrigel (50:50) solution were injected subcutaneously behind the fore limb of the 5-week-old mice using a 25-g needle. Tumors reached an average diameter of 6.4±0.6 mm prior to imaging experiments.

Optical imaging was performed with an IVIS spectrum small-animal *in vivo* imaging system (PerkinElmer) with integrated isoflurane anesthesia. This system consists of a cooled slow scan CCD camera and a light tight chamber that facilitates detection of very low light levels. A non-injected control animal was included. Images were acquired at regular intervals post injection with more frequent images taken at early time points and less frequent imaging thereafter. Images were taken using the settings of excitation 675 nm (30 nm band pass filter) and emission 720 nm (20 nm band pass filter) narrow band pass filters and were analyzed using Living Image Software v4.7.2 (PerkinElmer).

Fluorescent intensity is reported in units of radiant efficiency (radiance/incident excitation power) [(p/sec/cm2/sr) /(μW/cm2)] and total radiant efficiencies (TRE) [(p/sec)/(μW/cm2)] were calculated by drawing regions of interest on tumor and background. The tumor-to-background ratio (TBR) was calculated by using the measured TRE in the ROIs of tumor target and average background. Three different background ROIs of same area size were selected and signal averaged to give the mean signal background value.

Mice were injected via tail vein (2, 4 or 8 mg/kg based on PEG size) to give amounts as follows; [n = 4 for **1a** (3.7 nmol)], [n = 3 for **1b** (3.8 nmol)], [n = 3 for **1c** (3.4 nmol)], [n = 2 for **2** (1.9 nmol)], anesthetized, and imaged at various time points post injection.

All fluorescence images were acquired with two second exposure (f/stop = 2). All *in vivo* images are shown on the same color scale to illustrate change in contrast of target tumor region to background over time. Quantification is not affected by adjustment of the color scale.

## Results and Discussion

### Simulation of Tumor Fluorescence Time Profiles

**Figure 4:**
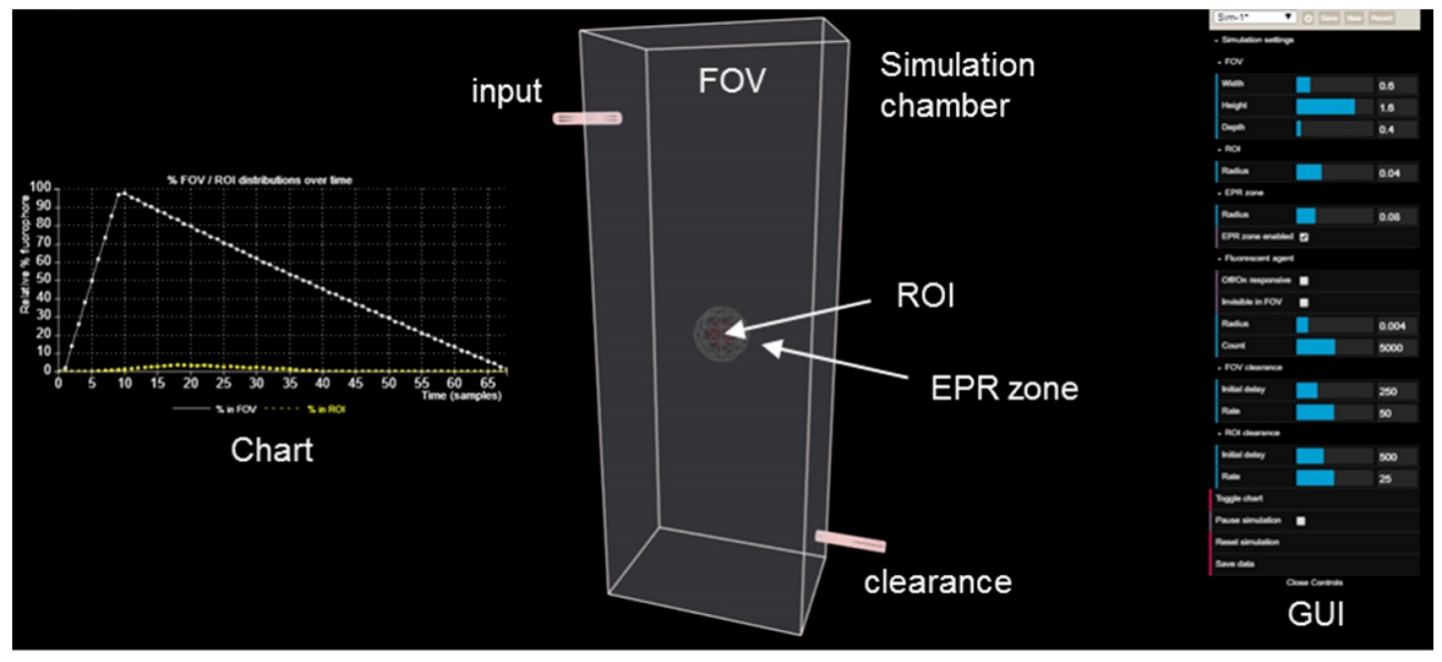
View of the simulation user interface showing simulation chamber FOV, tumor ROI, EPR zone, fluorophore input, clearance output, fluorophore distribution chart and GUI.

When designing new fluorescent imaging agents for *in vivo* use it is useful to consider the fact that the concentration of fluorophore in different tissues is continually changing over time and that identifying the optimal time at which an image is captured is essential. Moreover, in clinical imaging for FGS, this time point must coincide with the stage of surgery at which key clinical decisions need to be made. As such, a simulation model has been constructed to dynamically illustrate the influences on imaging of some pharmacokinetic parameters, the EPR effect, *always-on* fluorophores and bio-responsive *off-to-on* fluorophores (Figure 4). This simulation is qualitative in nature and valuable as a visual guide of the interplay between fluorophore bio-distributions and imaging, and can be useful in determining which characteristics may be most beneficial for clinical translation. As the simulation runs, the viewer can watch the changing contrast between surrounding normal tissue and tumor visualized in real-time from start to finish. The simulation interface permits a user to select and modify key factors such as the relative sizes of the normal tissue FOV to tumor ROI, clearance rates and an optional EPR zone of influence (Figure 4, Table 1).

Additionally, fluorophore characteristics such as quantity and relative size can be chosen and simulations can be run with optional modes of either *always-on* or *off/on* responsive fluorescence (Table 1). *Always-on* fluorophores are red in color, while *off-to-on* responsive fluorophores are shown as blue in the off state and red in the on state (after uptake into the ROI). Fluorophores in the off state may be selected to be invisible during the simulation, removing visual noise for the viewer without affecting the outcome of the simulation 40. Motion of fluorophores within the simulation chamber is programmed to follow random rectilinear trajectories and is not designed to mimic blood flow. The fluorophores do not interact with each other and, as soon as they happen to transit the EPR zone, their trajectory is gradually directed towards the ROI. Once inside the EPR zone, a fluorophore has a 50% chance of being released back into the FOV, if it happens to collide with the EPR zone surface. Initial delay periods (length of time in which no clearance takes place following start of the simulation) and rates of clearance from the FOV and ROI can be assigned independently, thereby modelling metabolic and excretion profiles of normal and cancerous tissues 41. Chart plots record the real-time change in percentage fluorophore distribution between the FOV and ROI as the simulation proceeds, allowing the user to semi-quantitatively compare the effects of the different parameters. Plot data can be exported in an Excel CSV format upon completion of the simulation. During development of this software, it became apparent that uses beyond our current focus of FGS in areas such as drug delivery, nanoparticle science, pharmacokinetics and chemical education are likely. Accordingly, access to the software is open sourced, allowing free use and modification of the code.

**Figure 5:**
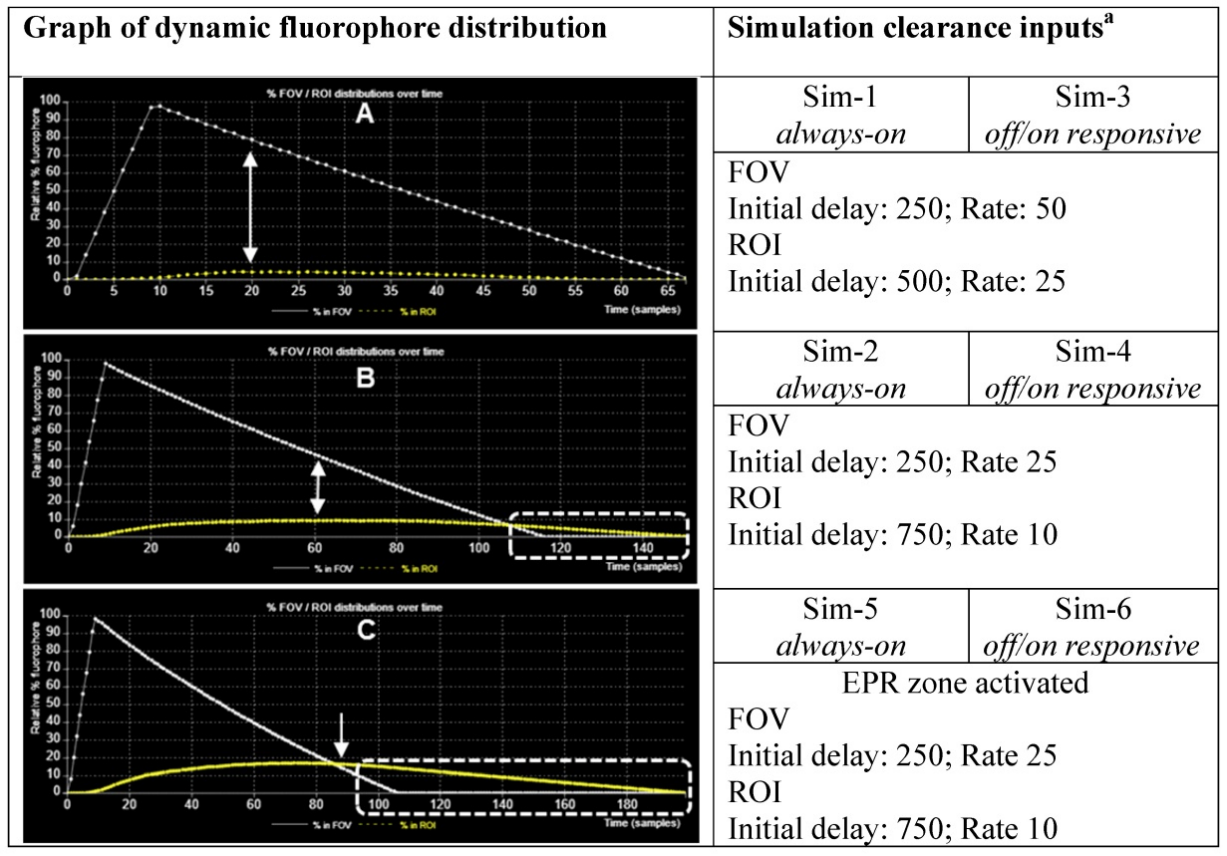
Chart of fluorophore uptake in ROI over time. (A) Plot showing % fluorophore distribution in the FOV and ROI over time with a fast clearance from both FOV and ROI. Arrows indicating point of maximum uptake into ROI and corresponding relative amount in the FOV. (B) Plot showing % fluorophore distribution in the FOV and ROI over time with a fast clearance from the FOV and slower clearance from ROI. Arrows indicating point of maximum uptake into ROI and corresponding relative amount in the FOV. Dashed highlighted area indicates full background clearance from the FOV has occurred with fluorophore remaining in the ROI. (C) Plot showing % fluorophore distribution in the FOV and ROI over time with a fast clearance from the FOV, a slower clearance from ROI and the EPR zone activated. Arrows indicating point where maximum uptake into ROI and corresponding relative amount in the FOV. Dashed highlighted area indicates full background clearance from the FOV has occurred with fluorophore remaining in the ROI. ^a^ FOV and ROI initial delay and rate values for in simulation are in relative au.

To illustrate points specifically related to this work a series of simulations (Sim 1-6) have been created which can be run at https://mihaitodor.github.io/particle_simulation/index.html and video recordings are included in the SI (Movie S1-S6). However, the reader is encouraged to run the simulations themselves with alternative parameters to observe their effects. Example simulations are included to demonstrate the impact of key features related to this work such as rates of clearance from normal and cancerous tissues, differences between *always-on* and bio-responsive *off-to-on* fluorophores and the EPR effect upon tumor uptake and clearance (Figure 5). Due to the random fluorophore motion, it should be noted that repeated simulation runs using the same inputs give very similar but non-identical data sets.

**Figure 6:**
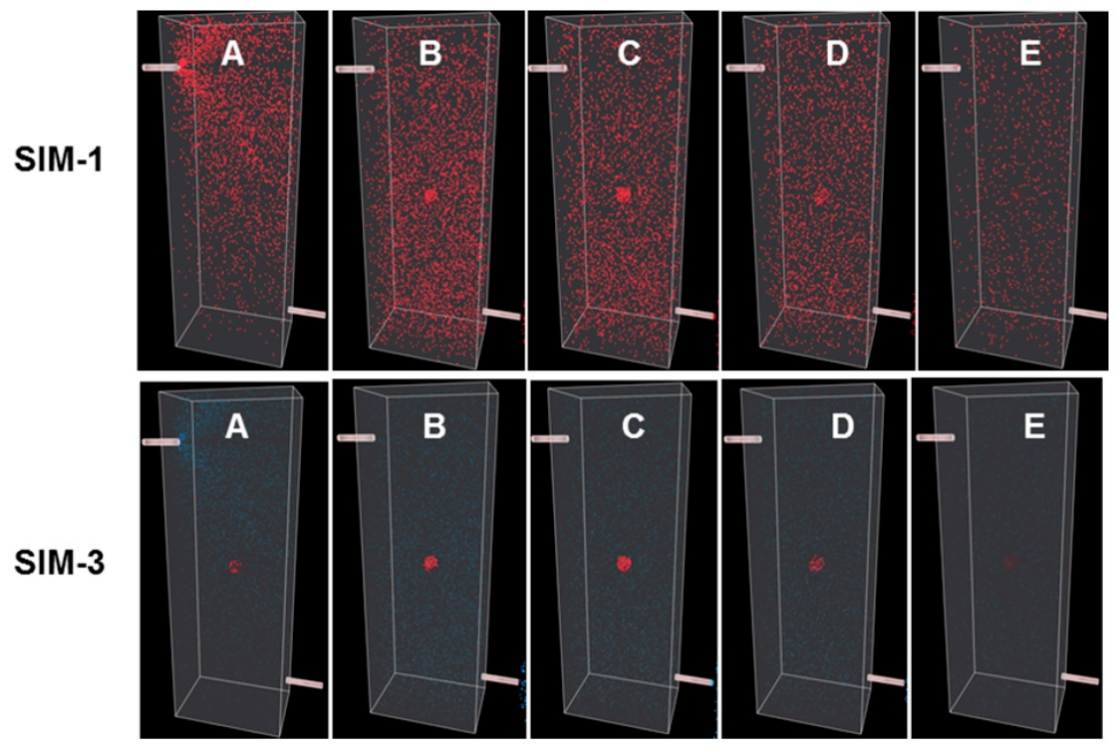
Simulations comparing *always-on* with *off-to-on* fluorophores with fast clearance rates from both FOV and ROI. **A** (start) to **E** (finish) corresponds to time intervals of 5, 10, 15, 35, 50 respectively from graph in figure 5A. Comparison of *always-on* fluorophore Sim-1 (top panel) and bio-responsive *off-to-on* fluorophore Sim-3 (bottom panel) at identical times points during the simulation. See Movies S1 and S3 for recordings of Sim-1 and Sim-3.

**Figure 7:**
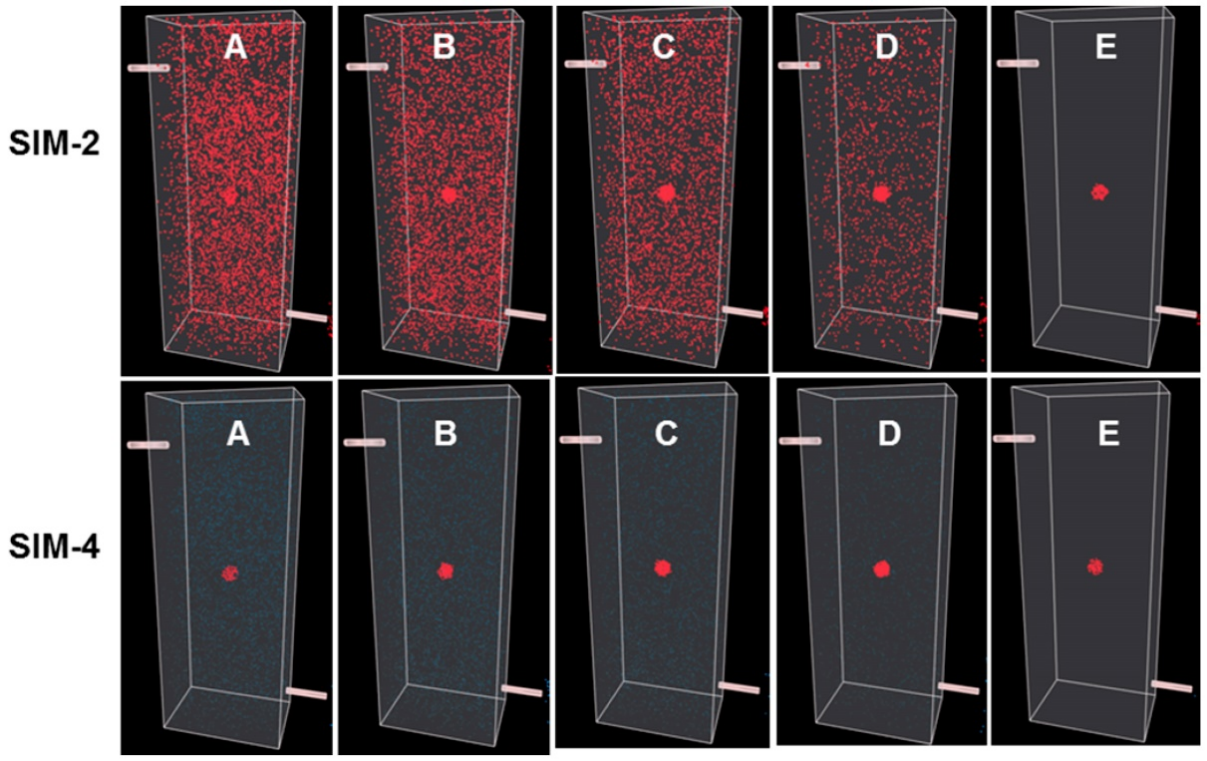
Simulations comparing *always-on* with bio-responsive *off-to-on* fluorophores with faster clearance rate from the FOV relative to the ROI. Still images **A** (start) to **E** (finish) correspond to time intervals of 10, 20, 40, 80, 115 respectively from graph in figure 5B. Comparison of *always-on* Sim-2 (top panel) and bio-responsive *off-to-on* Sim-4 (bottom panel) at identical times points during the simulation. See Movie S3 and S4 for recordings of Sim-2 and Sim-4.

Sim-1 and Sim-2 demonstrate the imaging challenge when utilizing an *always*-*on* fluorophore to identify the time point at which sufficient contrast for ROI imaging is achieved and how strongly dependent this is on the clearance rates from the FOV and ROI (Figure 6 and 7 top panels, Movies S1, S2). Inputs for Sim-1 and Sim-2 are identical for all settings except for FOV and ROI clearance rates. For Sim-1 input values for clearance of fluorophore from the FOV were chosen to simulate a fast clearance (settings: initial delay 250, rate 50) with the ROI given a moderately slower clearance rate (settings: ROI initial delay 500, rate 25). These values are representative of the clinically known very short half-life of ICG and do not provide sufficient contrast between normal and cancerous tissue at any time point 42. This is shown in the graphed results of Figure 5A, in which it can be seen that the maximum ROI fluorophore concentration is reached at a time when the majority of the fluorophore is in the background FOV (indicated by the arrows). As such insufficient contrast exists, which is confirmed upon viewing of Sim-1 where the observer can appreciate that at no time can the ROI be clearly distinguished from FOV background (Figure 6, top panel, SI Movie S1).

One current approach for improving contrast is to employ antibody-conjugated fluorophores that actively target cancer cells. In this case, it would be expected that the pharmacokinetic characteristics of the high molecular weight antibody would dominate that of the fluorophore, considerably extending the half-life within the vasculature and increasing uptake into the tumor. To simulate this, slower values for relative clearance rates for both FOV and ROI in Sim-2 were selected while maintaining more than a twofold faster clearance from FOV over ROI (settings: FOV initial delay 250, rate 25 and ROI initial delay 750, rate 10). Results from this simulation are shown in Figure 5B and Figure 7 (top panel) and can be viewed in SI Movie 2. This simulation shows that it takes a longer time for clearance from both ROI and FOV, but that the FOV fully clears first, leaving a period of time, highlighted by dotted white box, in which only the ROI has fluorophore thereby providing high contrast (Figure 5B). Importantly, this does indicate that prolonging the fluorophore within the vasculature should increase tumor uptake. The downsides to this approach are the lengthy waiting period for FOV clearance and the practical difficulties of routinely matching this with hospital surgical scheduling. In addition, as tumor clearance is ongoing, the risk exists that by the time of intraoperative imaging some tumor regions may no longer have sufficient fluorophore remaining.

The *off-to-on* responsive nature of the fluorophore is simulated by a color change from blue to red, indicating a switch from a non-fluorescent to fluorescent state. This is portrayed in Sim-3 and Sim-4 which have identical parameters to Sim-1 and -2 respectively except they are run in responsive mode (Figures 6 and 7, bottom panels). Fluorescence distribution plots for paired simulations Sim-1/3 and Sim-2/4 are essentially identical, but the differences are remarkable when the simulations are viewed (Movies S1-S4). Using Sim-4 as an example, the contrast provided by the *off/on* responsive mode allows the observer to distinguish the ROI at a very early stage of the simulation in spite of the fact that up to 90% of the fluorophore is in the FOV and only 10% in the ROI (Movie S4). As ROI can be identified for both Sim-3 and -4 once accumulation within the ROI begins, this illustrates how the bio-responsive fluorophore can overcome the reliance on background clearance kinetics to achieve imaging contrast (Figure 6, 7 bottom panels and Movies S3 and S4).

**Figure 8:**
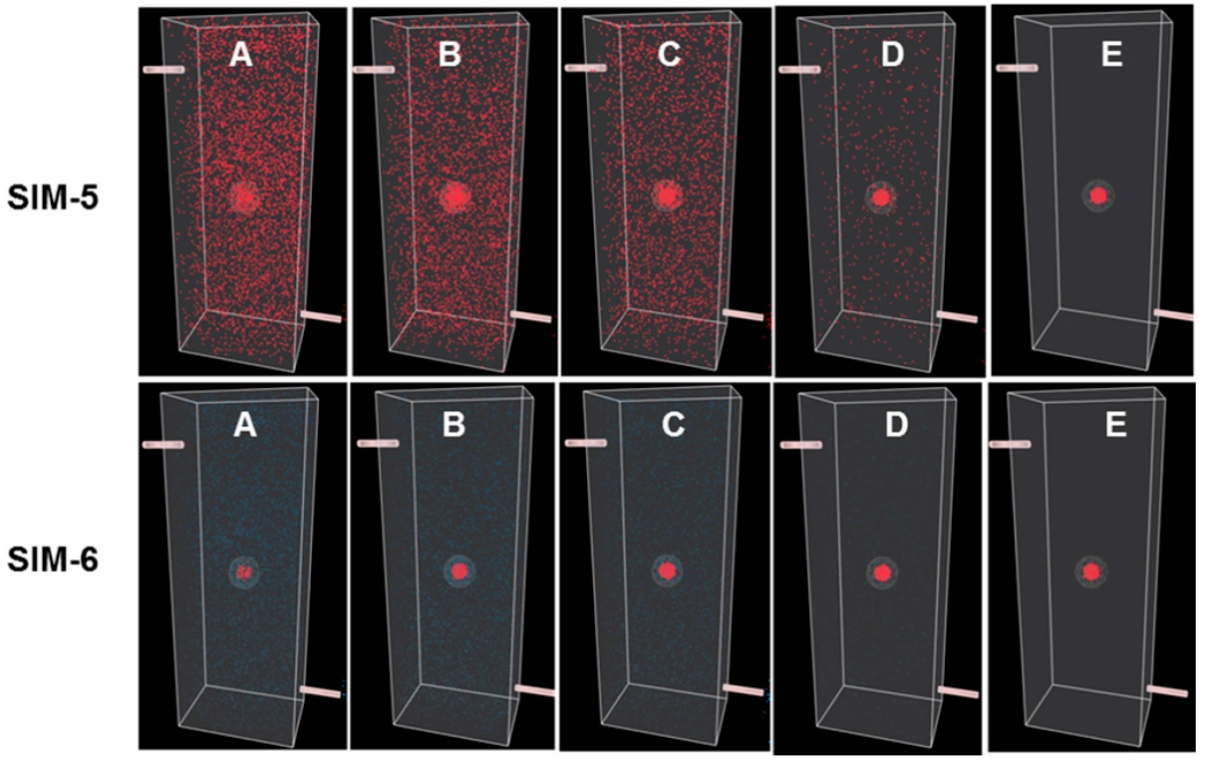
Simulations comparing *always-on* and *off-to-on* fluorophores with EPR zone enabled. Still images **A** (start) to **E** (finish) correspond to time intervals of 10, 20, 40, 90, 120 respectively from graph in figure 5C. Comparison of *always-on* Sim-5 (top panel) and bio-responsive *off-to-on* Sim-6 (bottom panel) at identical times points during the simulation. See Movie S5 and S6 for recordings of Sim-5 and Sim-6.

The next pair of simulations, Sim-5 and -6, were designed to demonstrate the EPR effect (Figure 8). This is achieved by including an outer EPR zone that accumulates and retains fluorophores that enter it, thereby statistically increasing the likelihood of ROI uptake. In addition, an ROI excretion rate considerably slower than that of the FOV was selected to model the retention associated with the EPR effect. These parameters allow a graphic and visual representation of the effect on temporal fluorophore distribution by the leaky vasculature and poor lymphatic drainage associated with a tumor.

Sim-5 has the same FOV and ROI clearance settings (FOV initial delay 250, rate 25 and ROI initial delay 750, rate 10) as SIM-2 but with the EPR zone enabled (option selected from GUI) (Figure 8). The graphed results in Figure 5C show the increased ROI uptake relative to Sim-2.

Encouragingly, the time point at which the ROI and FOV have equal fluorophore concentrations coincides with a maximum concentration for the ROI, as indicated by the single headed arrow (Figure 5C). It is noteworthy that the intersection point of equal FOV and ROI fluorophore concentrations occurs at an earlier time point than Sim-2 and once clearance is complete from the FOV a longer period is available in which optimal contrast would be available for imaging (Figure 5c, dashed highlighted area). This simulation indicates that the advantage of exploiting the EPR effect could be three fold: elevated fluorophore concentration in the tumor, contrast achieved at an earlier time point and persistence of good contrast for a more prolonged time. The bottom panel in Figure 8 shows the time sequence of images from Sim-6, recordings of which can be seen in Movie S6. This simulation is run with the fluorophore in responsive mode, thereby providing an enhanced ROI contrast soon after introduction of fluorophores into the chamber (Figure 8, bottom panel). For comparison, SI Movie S5 shows the same simulation run in *always-on* fluorophore mode in which it is not possible to distinguish the ROI at such an early stage. As our simulation results indicated a strong potential for an EPR imaging approach, several *off/on* and *always-on* NIR-AZA fluorophores with different sized PEG substituents were synthesized for testing.

### Preparation of PEG conjugated NIR-AZAs 1a-c and 2a

**Scheme 1:**
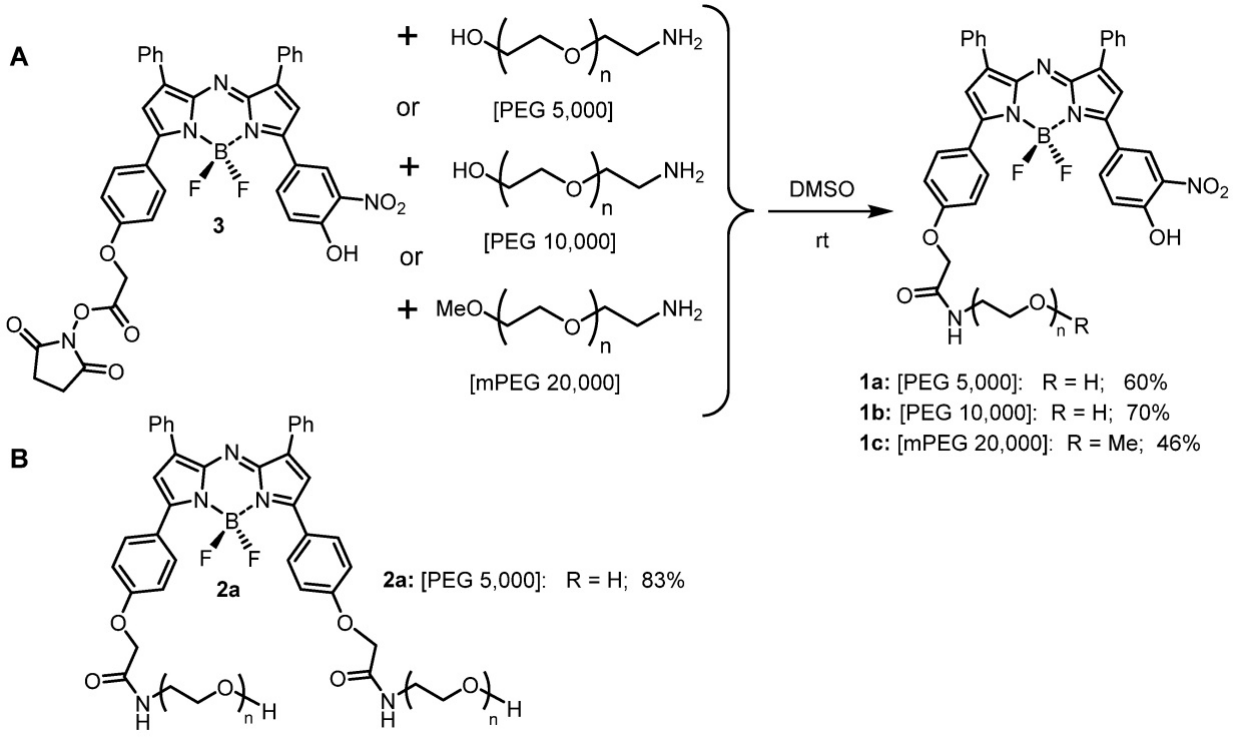
NIR-AZA fluorophores used in this study. (A) Pegylation of NIR-AZA **3** to produce **1a**-**c**. (B) structure of *always-on* NIR-AZA **2a**.

Three heterobifunctional amino-substituted PEGs with molecular weights of 5, 10 and 20 kDa were selected to provide a distribution of mass sizes. Conjugation reactions were performed in DMSO, utilizing the activated ester substituted NIR-AZA **3** (1.2 eq.) 38 with the corresponding amino-PEG (1 eq.) proving effective under rt conditions (Scheme 1).

A small excess of activated ester **3** was used to ensure full consumption of all of the PEG reagent and upon reaction completion after 6 h, the crude products were purified using semi-preparative HPLC (Figure S1). Post purification, **1a**-**c** were obtained in 60%, 70% and 46% yield respectively. ^1^H NMR data and matrix-assisted laser desorption/ionization (MALDI) mass analysis for conjugates showed the expected resonances and masses (Figure S2). An *always-on* pegylated derivative **2a** was included in this study to allow a direct a side-by-side comparison with *off/on* responsive **1a**-**c**. NIR-AZA **2a** containing two 5 kDa PEG groups was chosen as it has intermediate PEG molecular weight of 10 kDa and was synthesized following literature procedures (Scheme 1) 39.

As the EPR effect of pegylated molecules is related to their overall size dynamic light scattering measurements (DLS) were taken for the amino-PEG reagents used and their corresponding conjugates **1a**-**c** and **2a** in phosphate buffered saline (PBS). As anticipated, each of the amino-PEG reagents had diameter sizes less than 10 nm but once conjugated to a NIR-AZA fluorophore the size ranged between 100 and 300 nm (Table 2 entries 1-7). This can be attributed to the self-assembly of individual PEG conjugates into nanoparticles due to the amphiphilic nature of the conjugate, which has previously been observed for pegylated small molecule drugs such as doxorubicin 43. Notably, the nanoparticle assembles for **1a**-**c** and **2a** were all stable at 37 °C, indicating that this could contribute to their ability to induce an EPR effect (Table 2, entries 8-11).

### *Always-on* and *off/on* emission properties of 2a and 1a-c

**Figure 9:**
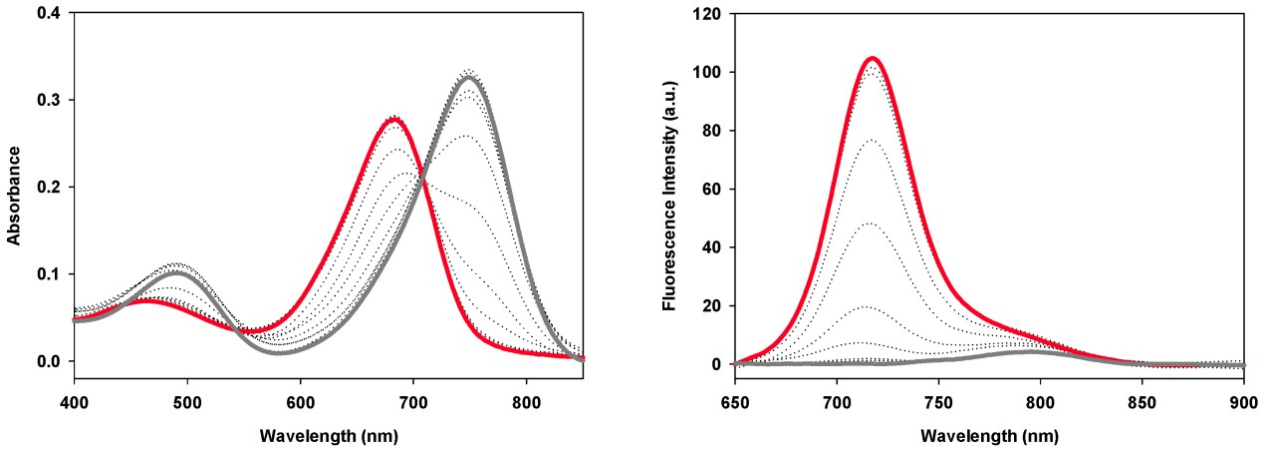
Absorbance (left) and fluorescence (right) spectra of **1b** (5 μM) in PBS buffer/TX-100 (0.34 mM) starting at pH 8 (grey line) to pH 2 (red line). Fluorescence excitation: 630 nm; range: 650 - 900; slit widths: 5/5. See ref 38 for plots of **1a** and Figure S3 for plots of **1c**.

The fluorescence switching properties of the PEG conjugates in response to pH were examined in a series of acid-base titrations in PBS. The absorbance properties of conjugates **1a**-**c** were very similar, with the phenolate state having a λ_max_ of 749±1 nm and the corresponding phenols having λ_max_ of 684±1 nm (Figure 9, Table 3). Each derivative showed weak ICT emissions centered between 789-796 nm at pH 8 that disappeared upon acidification of the solution. As the solution pH was sequentially lowered, stronger emission bands with λ_max_ of 716±1 nm were observed for each conjugate (Figure 9, Figures S3). The fluorescence enhancement factor for **1a**-**c** were above 20 and the pKa values of 4.6, 4.2 and 4.7 respectively were in close agreement with each other. In contrast, the *always-on* derivative **2a** showed no pH dependent changes in absorption or emission 39.

### *In vitro* live cell imaging in MDA-MB 231 cancer cell line with 1a-c and 2a

**Figure 10:**
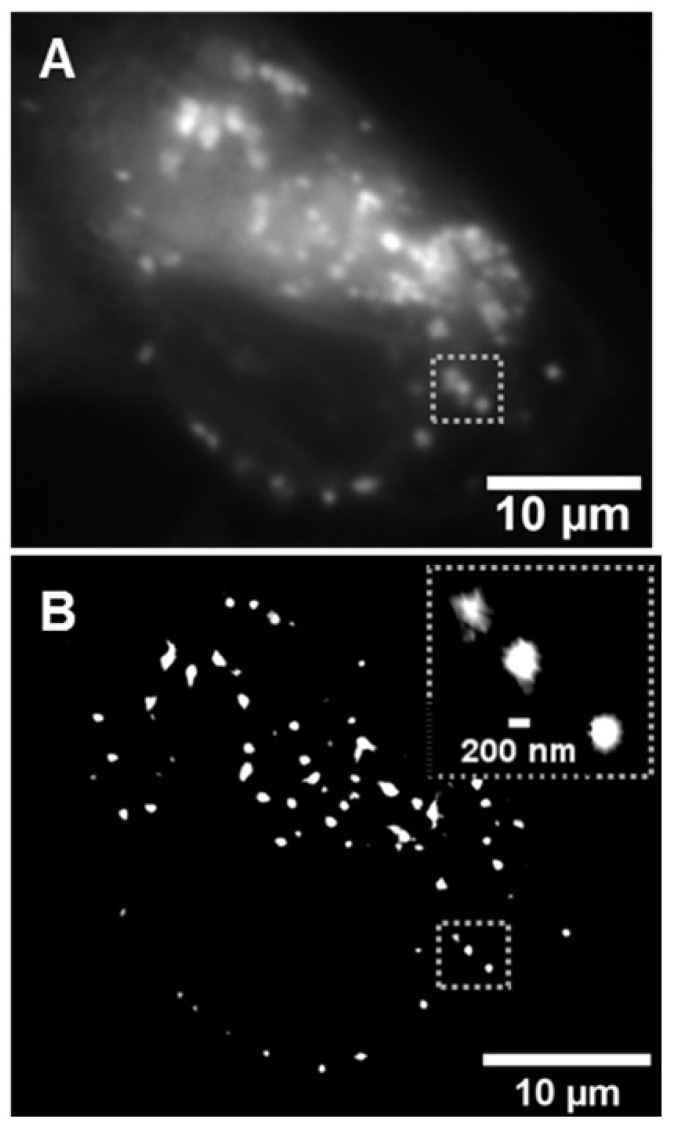
Image of lysosomal turn on of **1b** within MDA-MB 231 cells. (A) Widefield image following 2 h incubation with **1b**. (B) Super-resolution radial fluctuations (SRRF) image of the same cell following 2 h incubation with **1b**. Inset dashed box shows expansion of lysosomes within a small subcellular region.

The use of **1a** for live cell lysosomal imaging in HeLa cells has previously been reported 37. In this work **1a-c** were examined in the human breast cancer cell line MDA-MB 231, utilizing both widefield and super-resolution radial fluctuations (SRRF) imaging 44. As expected, following treatment of cells with **1a**-**c** no fluorescence was initially observed and over time a steady increase in fluorescence intensity was recorded as fluorophore entered the cell and switched on in the lysosomes (Figure 10A, and Figure S4). The uptake within cells was efficient, with a notable intracellular emission within 15 min and a steady increase in brightness over the following 100 min. Images acquired using super-resolution radial fluctuations (SRRF) methods, following 2 h incubation with **1b**, confirmed the strong emission preference to be from lysosomes (Figure 10B). These results are consistent with previously reported widefield imaging for **1a** 37. The next stage was to investigate if this intracellular switch on of fluorescence would be translatable to useful tumor imaging *in vivo*.

### Time profiles of* in vivo* tumor imaging with 1a-c and 2a

**Figure 11:**
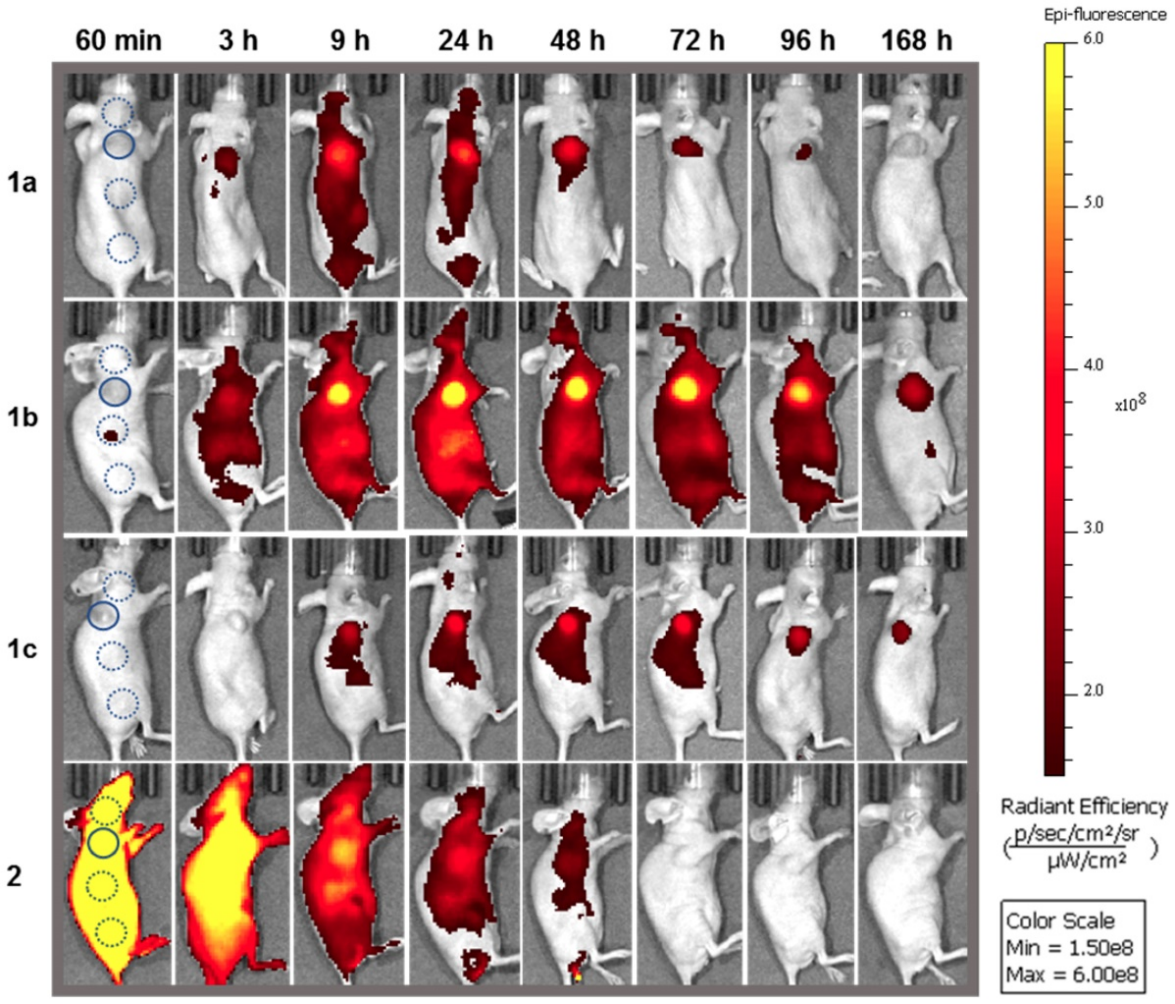
Analysis of fluorescence images for *off/on* responsive **1a**-**c** and *always-on* **2a** over 7 days. Representative *in vivo* fluorescence images of each using a MDA-MB 231 subcutaneous tumor model at different time points, all on the same radiant efficiency scale. First image shows selected tumor ROI (solid circle) and three background ROIs (dashed circles).

The imaging performance of pegylated bio-responsive conjugates **1a-c** and *always-on* derivative **2a** were tested using MDA-MB 231 derived subcutaneous tumors grown in nude mice (Figure 11). This human cancer cell line gives rise to an aggressive form of triple negative breast cancer, which is often first treated by surgery.

As the *in vivo* perfusion of contrast agents is a dynamic process from administration to excretion, fluorescence images were acquired over one week in the expectation that a holistic analysis of how the fluorescence distributions emerge, evolve and decline would be more revealing than focusing on a single static moment in time. It was anticipated that measurement of the fluctuating TBR for **1a-c** would identify which PEG group provides the best contrast for the longest time. In addition, if fluorescence turn on was preferentially biased to the tumor, this could provide a unique insight into the dynamics of the EPR effect for the different sized PEG groups. NIR-AZA **2a** was included in the study as an *always-on* control and to allow an experimental comparison with the simulations SIM-1, -2, and -5 in which the view of the ROI is obscured at the outset when using a non-responsive fluorophore.

**Figure 12:**
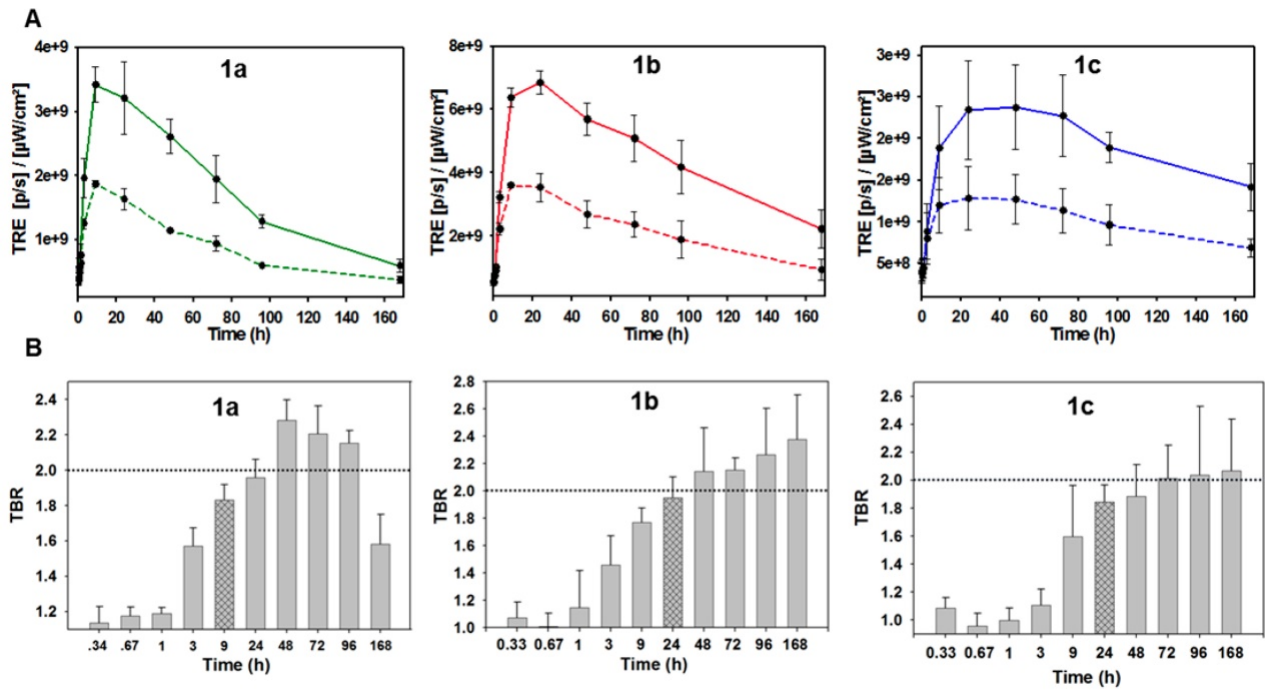
Temporal analysis of *in vivo* fluorescence distributions for **1a**-**c**. (A) Time course of measured total radiant efficiency (TRE) from tumor ROI (solid lines) and background (dashed lines) for **1a**-**c** over 7 days. (B) TBR analysis of *in vivo* fluorescence imaging for bio-responsive PEG NIR-AZA **1a** (n=4)^46^, **1b** (n= 3), **1c** (n=3) over 7 days. Dashed red line indicating threshold value of 2. Crossed bar in each plot indicates time of maximum tumor emission intensity. Values determined by ROI total fluorescence signal of tumor divided by an averaged value of three background regions as measured by Living Image Software v4.7.

Fluorophores **1a** (3.7 nmol),** 1b** (3.8 nmol), **1c**, (3.4 nmol) and **2a** (1.9 nmol) in PBS were administered by i.v. tail vein injection. Post injection, images were acquired frequently between 10 min and 24 h and thereafter at 48, 72, 96 and 168 h which are shown in Figure 11, all on the same scale of radiant efficiency. TBR values for all experiments were calculated by ratioing tumor ROI intensity against an average of three equally sized background ROIs, two of which were near to and one further away from the tumor (Figure 11, circled areas in first image). A TBR threshold value of 2 was used to compare different fluorophores as thresholds at or above this value have been reported to be of clinical relevance 45. The time taken to reach this value and the duration for which it is maintained were used to make cross comparisons for the different PEG conjugates. Plots of tumor and background radiance efficiency and TBR data over 7 days for **1a**-**c** are show in Figure 12A and 12B respectively 46.

Analysis of the background intensity data shows that fluorescence remained off for the first hour following administration proving the robustness of phenolate/phenol switch (Figure 12A, graphed dashed traces). This ability to suppress fluorescence from the outset highlights the key advantage of using *off-to-on* switchable fluorophores. While background fluorescence did increase over the next 24 h for **1a**-**c**, it is remarkable that at all times over the seven days, the intensity of fluorescence from the tumor was always higher than that of the background (Figure 12A. A study of tumor ROI radiance efficiencies for **1a**-**c** showed that they remained very low for the first 60 min with some increase at 3 h, but then rose rapidly with a maximum reached at 9 h for **1a** and 24 h for **1b** and **1c** (Figure 12A, graphed solid traces). At their maxima, the measured relative tumor emission intensities showed that **1b** > **1a** > **1c** with **1b** being 1.5 fold higher than **1a** and 3 fold higher than **1c**. This positive imaging impact from suppression of background fluorescence with turn on in the ROI is evident if the simulations Sim-5 and -6 are compared, which have the same distribution of fluorophore over time but with one being *always-on* (SIM-5) and the other being *off-to-on* responsive (SIM-6).

The relative decrease of tumor fluorescence from the time of maximum emission to 168 h was calculated as an indicator of enhanced retention due to the PEG substituents. For **1a**, a 7.7 fold intensity reduction occurred between 9 and 168 h, with near complete clearance within the experimental timeframe. For **1b** only a 3.1 fold reduction in intensity occurred, whereas **1c** had the smallest 1.6 fold reduction. The greatest reduction for **1a** is consistent with it being the lowest molecular weight PEG conjugate with the fastest excretion thereby reducing the quantity in circulation that would be available for tumor uptake. The slowest excretion rate was consistent with the highest molecular weight PEG of **1c**, whereas the intermediate 10 kDa PEG **1b** gave the best balance between tumor uptake and retention. It is important to put this data in a broader context of relative tumor brightness for the three derivatives **1a**-**c**. The higher tumor fluorescence intensity for **1b** over **1a** and **c** indicates that the enhanced uptake and retention aspects of the EPR effect are both working in its favor for imaging (Figure S5). This is not to say that **1a** and **1c** are not influenced by the EPR effect just that their rates of uptake and clearance differ from **1b** and are less favorable for imaging. Taken together these results evidently show how the molecular weight size can be used to fine tune the tumor uptake and excretion profiles to provide good imaging contrast, with **1b** being superior to **1a** and **c**.

Analysis of the temporal changes in TBR was equally revealing. For each derivative, the TBR remained low for the first three hours and thereafter steadily increased over the following 21 h, which coincided with rising tumor emission intensity. Using **1b** as an example, between 1 and 24 h the TBR improved from 1.1 to 2 with a simultaneous 6.6-fold increase in tumor fluorescence intensity. Additionally, the TBR continued to increase for the following six days despite decreasing tumor and background emission intensities indicating that tumor clearance rates are slower than background. This combined analysis of fluorescence intensity and TBR profiles provides evidence that enhanced tumor accumulation and retention of **1b** is PEG mediated due to the EPR effect. The largest molecular weight 20 kDa PEG derivative **1c** showed the poorest imaging performance with lowest fluorescence intensity (Figure 11). This can be rationalized by its relatively prolonged retention, with fluorescence switched off, within the vasculature. The distribution profiles for **1c** would appear to be better suited to drug delivery for which an extended and sustained tumor uptake is more desirable, whereas reaching the highest concentrations as quickly as possible is preferable for imaging. Evidence of PEG group influencing tumor retention is also apparent at the last 168 h imaging time point as both **1b** and **1c** sustained a TBR above 2 whereas **1a** dropped below this threshold after 96 h, which is in accord with its lower molecular weight (Figure 12B).

**Figure 13:**
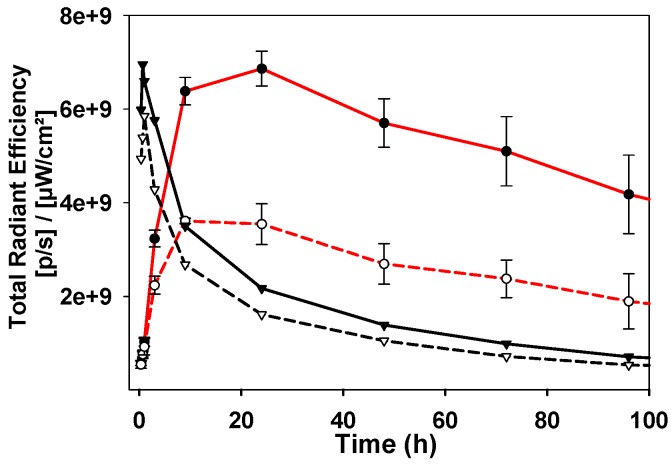
Comparison of emission intensities from tumor (solid lines) and background (dashed lines) for **2a** (black traces) and **1b** (red traces).

The image sequence for *always-on* **2a** is strikingly different from those of **1a-c** with fluorescence intensity at a maximum immediately after administration (Figure 13). There is a steady decrease from peak intensity, though little divergence of plot lines for tumor emission (Figure 13, black solid trace) and background (Figure 13, black dashed trace), which is consistent with the TBR for **2a** never reaching the threshold value (Figure S6). This profile can be related to that seen in SIM-1 in which no clear discrimination of tumor from background is achieved (Movie S1). As a side-by-side comparison the emission profiles of responsive **1b** are included on the same plot, which start at very low intensity, increase over 24 h and then decrease over the following 6 days (Figure 13, red traces).

**Figure 14:**
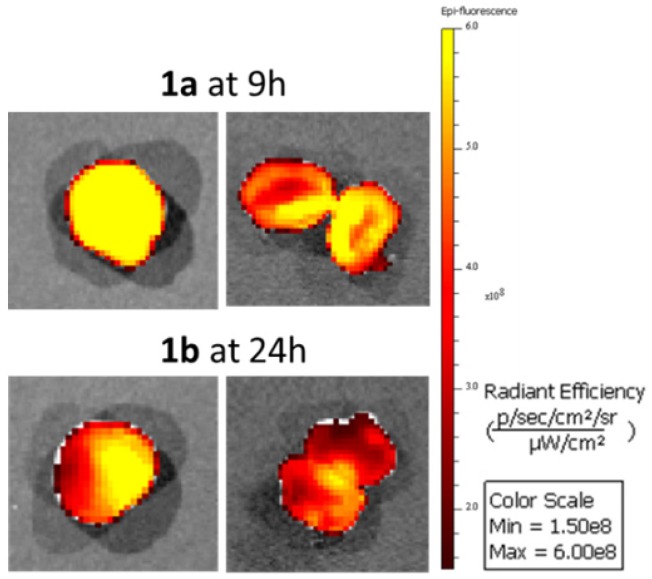
Excised and dissected tumors from animals treated with **1a** for 9 h and **1b** for 24 h.

The time points of maximum tumor emission for **1a** and **1b** were identified as 9 and 24 h respectively so in additional experiments tumors were resected from animals at these times for direct imaging (Figure 14). In both cases confirmation of tumor labelling was achieved as tumors were strongly fluorescent and upon dissection, the fluorescence was throughout but brighter at the outer margins. Also seven days following administration of **1c** of animals low levels of tumor fluorescence was detectable following resection (Figure S7). A comparison of fluorescence intensity from tumors and excised organs for animals treated with **1a** for 9 h and **1b** for 24 h is shown in Figure S8. This data confirmed that tumors had the highest average fluorescence for both fluorophores at these time points.

## Conclusions

A wealth of evidence indicates that tumor blood vessels differ significantly from normal vessels in their structural organization and that this can be exploited for improved drug delivery by the pegylation of drug molecules. In this report, we have investigated the use of NIR-fluorophore pegylation in an effort to gain advantage from the EPR effect for *in vivo* fluorescence imaging with the goal of translation to FGS.

To assist in conceptualizing the challenge at hand, an imaging simulation model was developed to illustrate dynamic influences of fluorophore temporal distribution, the EPR effect 47, *always-on* and bio-responsive fluorophores on imaging. The freely available software allows a user to input key fluorophore, metabolism and EPR parameters and to observe the influence on imaging performance in real-time, providing relative ROI and FOV quantification data. To progress this concept, three bio-responsive NIR-AZA fluorophores 48 have been synthesized and characterized with varying sized PEG attachments. Each responsive derivative showed excellent off/on excited state control with large fluorescence enhancement values. Super resolution fluorescence imaging in MDA-MB 231 cells showed an effective switch on upon cell uptake localized within the lysosomes. A comparative *in vivo* assessment of tumor imaging performance for bio-responsive **1a**-**c** and *always-on* derivative **2a** was conducted with recording of background and tumor fluorescence strengths over 168 h. The fidelity of the phenolate to phenol switching was maintained for **1a**-**c** as they remained fluorescent silent within the vasculature for the first hour following i.v. administration. Tumor accumulation and retention was most effective for **1b**, with a TBR value of **2** reached by 24 h and maintained until the end of the study at 168 h. While compounds **1a** and **1c** have similar overall profiles they did not perform as well as **1b** as lower molecular weight **1a** cleared too quickly and **1c** had limited tumor uptake. Cross comparison of the data for all three responsive derivatives showed evidence of the EPR effect and that it could be optimized for tumor imaging through identification of the optimal PEG group. As anticipated, non-responsive **2a** has very high intensity immediately upon administration and did not clearly reveal the tumor at any time point.

The next phase of our work is to gather continuous kinetic data on the tissue dependent rates of emission increase over time and computationally mine this data stream to developed AI algorithms for dynamic image analysis. This could provide future surgical team with an augmented reality map of the extent of cancerous growth within the normal tissue during the operation. As tumor-free surgical margins are critical to the success of cancer surgery, new boundary revealing technologies could have far-reaching benefits for cancer patients.

## Supplementary Material

Supplementary figures and movie legends.

Supplementary movie 1.

Supplementary movie 2.

Supplementary movie 3.

Supplementary movie 4.

Supplementary movie 5.

Supplementary movie 6.

## Figures and Tables

**Table 1:**
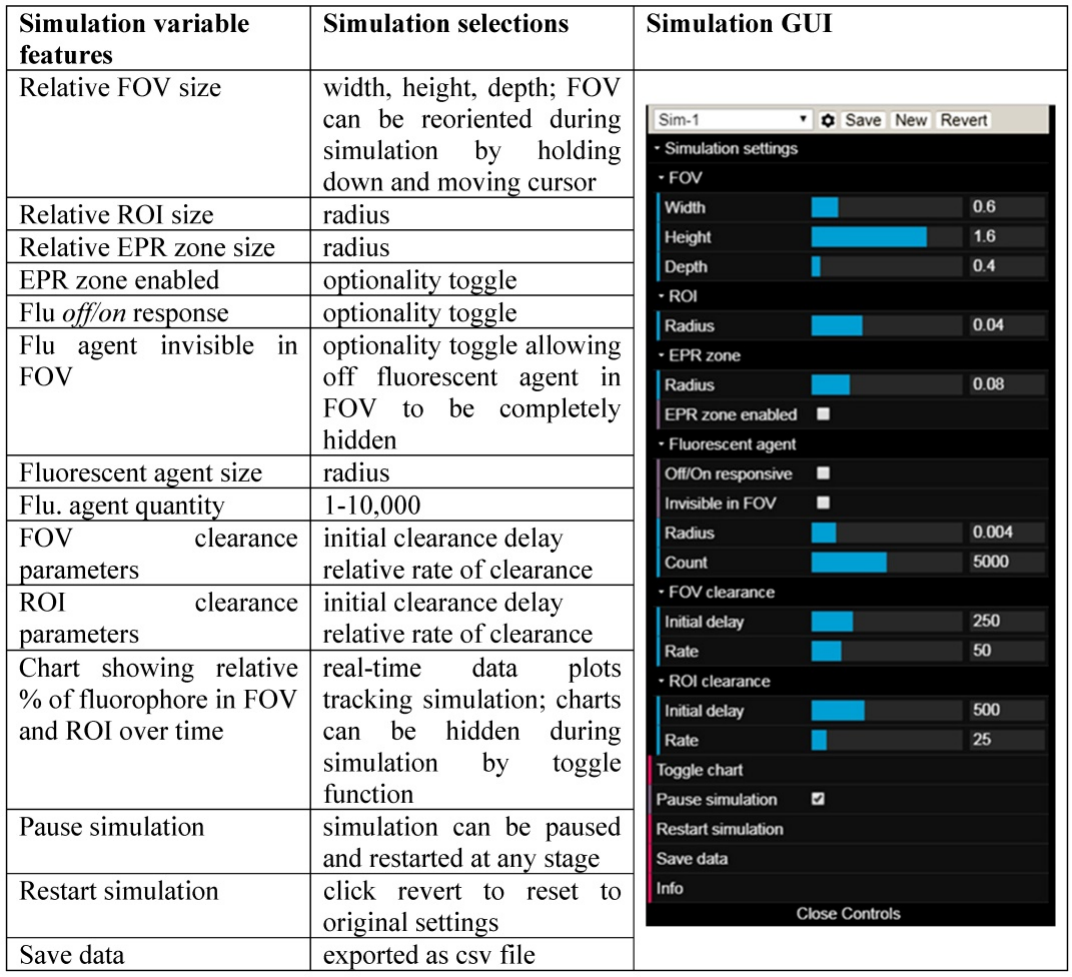
Simulation variables and graphical user interface (GUI).

**Table 2:** DLS data for PEG reagents and conjugates **1a**-**c**, **2a** in PBS.^a^

Entry	compound	Temp °C	Size (nm)	PDI
1	PEG 5,000	20	2.4±0.3	0.34
2	PEG 10,000	20	5.8±1.4	0.56
3	PEG 20,000	20	9.7±0.5	0.35
4	**1a**	20	106±9	0.52
5	**1b**	20	302±22	0.35
6	**1c**	20	258±14	0.47
7	**2a**	20	343±40	0.40
8	**1a**	37	216±45	0.65
9	**1b**	37	343±12	0.17
10	**1c**	37	170±10	0.27
11	**2a**	37	210±15	0.40

a. Measurements taken at a concentration of 5 µM.

**Table 3:** Off/on switching characteristics of **1a**-**c** and always-on **2a**.^a,b^

Entry	compound	λ_max_ abs (nm) pH 8 /2	λ_max_ flu (nm) pH 8 /2	FEF^c^	pKa
1	**1a**	749/684	790/716	20.1	4.6 38
2	**1b**	749/684	796/718	22.2	4.2
3	**1c**	750/683	789/716	25.9	4.7
4	**2a**	690/690	722/722	0	-

^a^5 µM in PBS. ^b^ To allow a common comparison for each fluorophore across a wide pH range 0.34 mM Triton X-100 was included in each solution. Fluorescence enhancement factor (FEF) measured integrated fluorescence differences at 7.4 and 4.5.
