# Supplementary material for: An EPR Strategy for Bio-responsive Fluorescence Guided Surgery with Simulation of the Benefit for Imaging
Source: Theranostics. 2020 Feb 10;10(7):3064–82. doi: 10.7150/thno.42702 (PMC7053210; doi:10.7150/thno.42702)

An EPR Strategy for Bio-responsive Fluorescence Guided Surgery with Simulation of the Benefit for Imaging

Harrison C. Daly,[1] Emer Conroy,[2] Mihai Todor,[1] Dan Wu,[1] William M. Gallagher,[2] Donal F. O'Shea*[1]

[1] Department of Chemistry, RCSI, 123 St. Stephen's Green, Dublin 2, Ireland.

[2] School of Biomolecular and Biomedical Science, Conway Institute, University College Dublin, Belfield, Dublin 4, Ireland.

Supporting Information

Legends for Movies S1 - S6 S2

Figure S1: HPLCs of **1a-c**, **2a** S3

Figure S2: NMR of **1a-c**, **2a** S5

Figure S3. Photophysical spectra of **1c** S7

Figure S4. Widefield fluorescence MDA-MB 231 cells images of **1a-c** S8

Figure S5. Plot of tumor emission intensities over time for **1a-c** S9

Figure S6. TBR analysis for *always-on* PEG NIR-AZA **2a** S10

Figure S7. Fluorescence image of excised tumor from animal treated with **1c** S10

Figure S8. Fluorescence intensity of tumor and excised organs for **1a** and **1b** S11

Legends for Movies of Simulations

Movie S1: Simulation showing imaging with fast clearance rates from both FOV and ROI with fluorescence in *always-on* mode (Sim-1).

Movie S2: Simulation showing imaging with faster clearance rate from the FOV relative to the ROI with fluorescence in *always-on* mode (Sim-2).

Movie S3: Simulation showing imaging with fast clearance rates from both FOV and ROI with fluorescence in *off-to-on* mode (Sim-3).

Movie S4: Simulation showing imaging with faster clearance rate from the FOV relative to the ROI with fluorescence in *off-to-on* mode (Sim-4).

Movie S5: Simulation showing imaging with faster clearance rate from the FOV relative to the ROI and EPR zone enabled with fluorescence in *always-on* mode (Sim-5).

Movie S6: Simulation showing imaging with faster clearance rate from the FOV relative to the ROI and EPR zone enabled with fluorescence in *off-to-on* mode (Sim-6).

Figure S1. HPLC traces for **1a-c** and **2a**

HPLC trace of **1a**

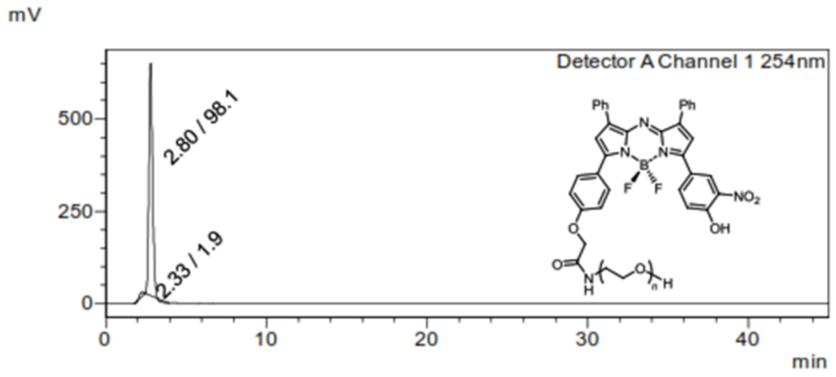

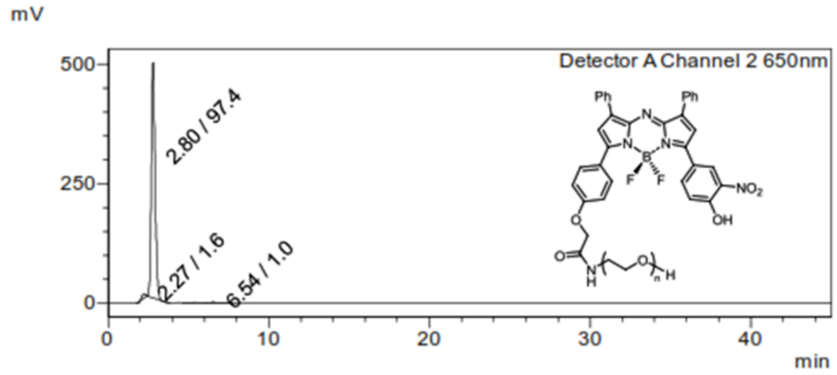

HPLC trace of **1b**

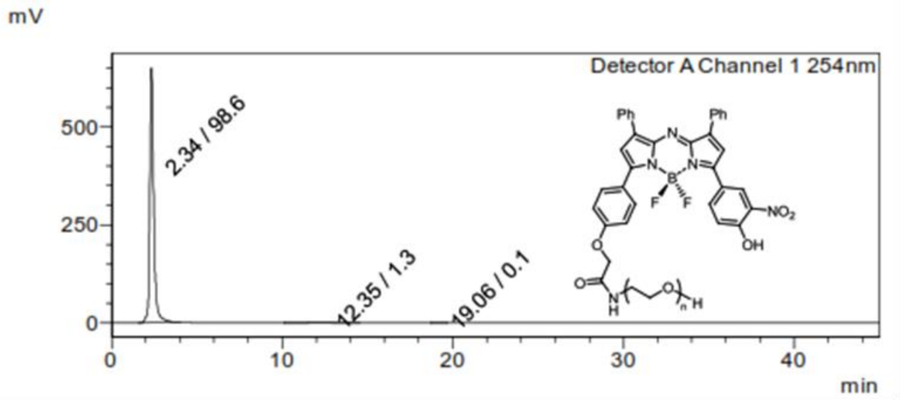

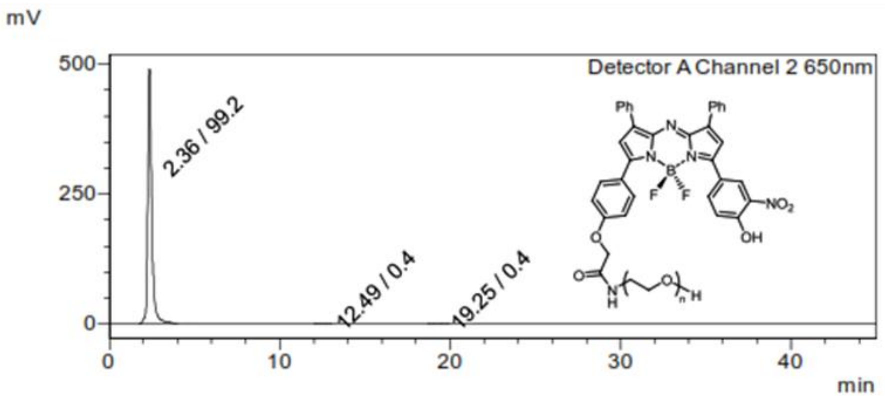

HPLC trace of **1c**

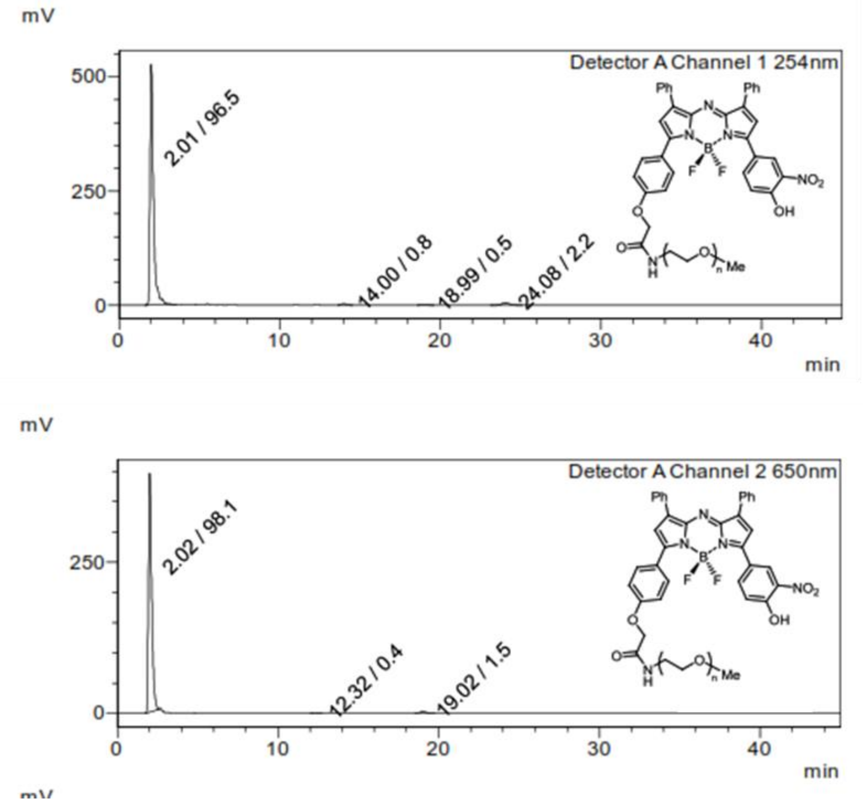

Condition: RP-HPLC with YMC triart phenyl column. Detection method: UV-Vis wavelengths: 254 nm and 650 nm. Eluent CH_3CN:H_2O =60:40 with 10 mM NH_4HCO_3.

HPLC trace of **2a**

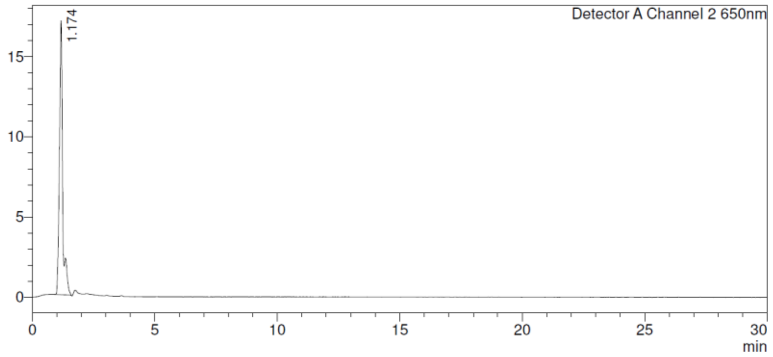

Condition: RP-HPLC with YMC triart phenyl column. Detection method: UV-Vis wavelength 650 nm. Eluent CH_3CN:H_2O =70:30.

Figure S2. NMR Spectra of **1a-c** and **2a**

^{1}H NMR of **1a**

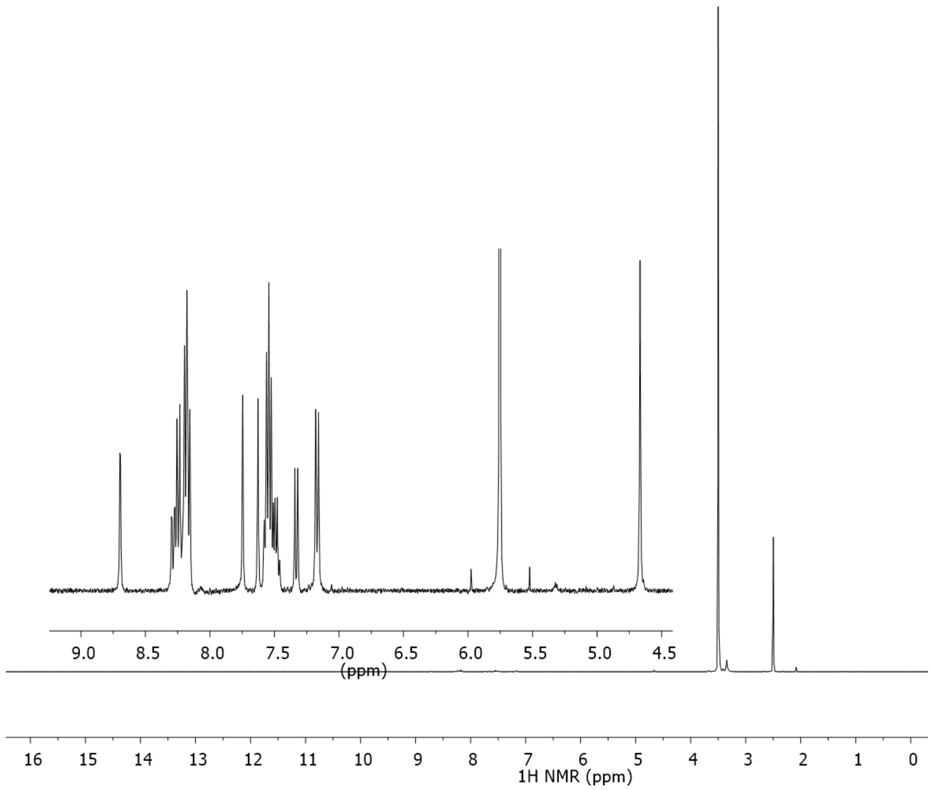

^{1}H NMR of **1b**

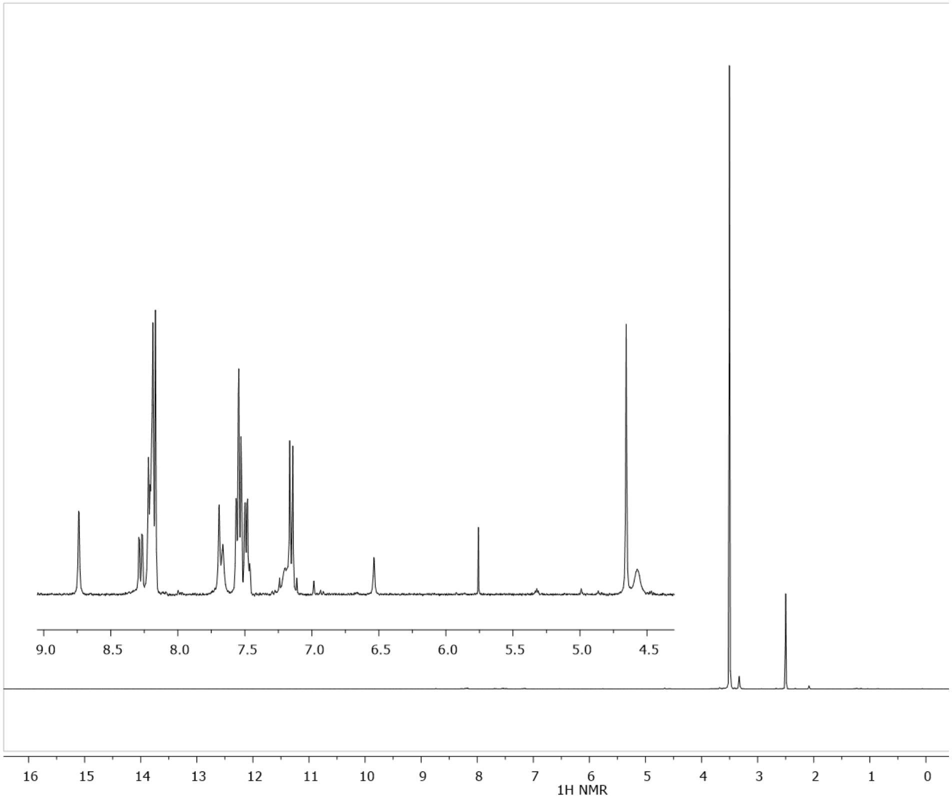

^{1}H NMR of **1c**

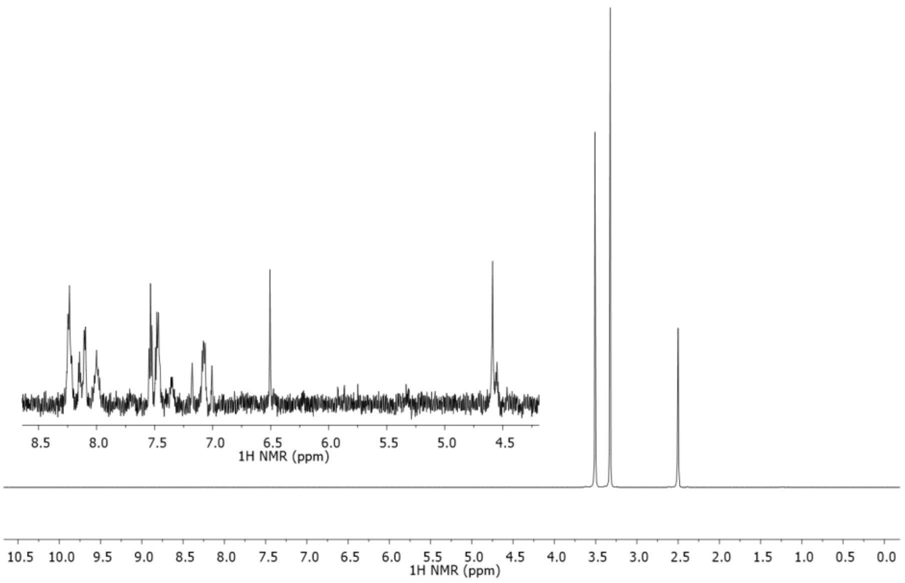

^{1}H NMR of **2a**

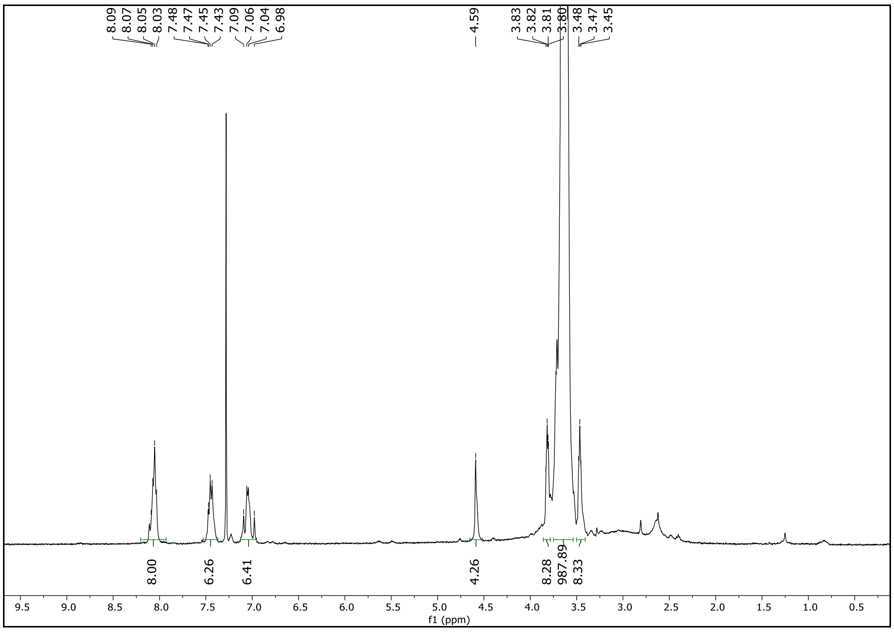

Figure S3: Photophysical properties of **1c**

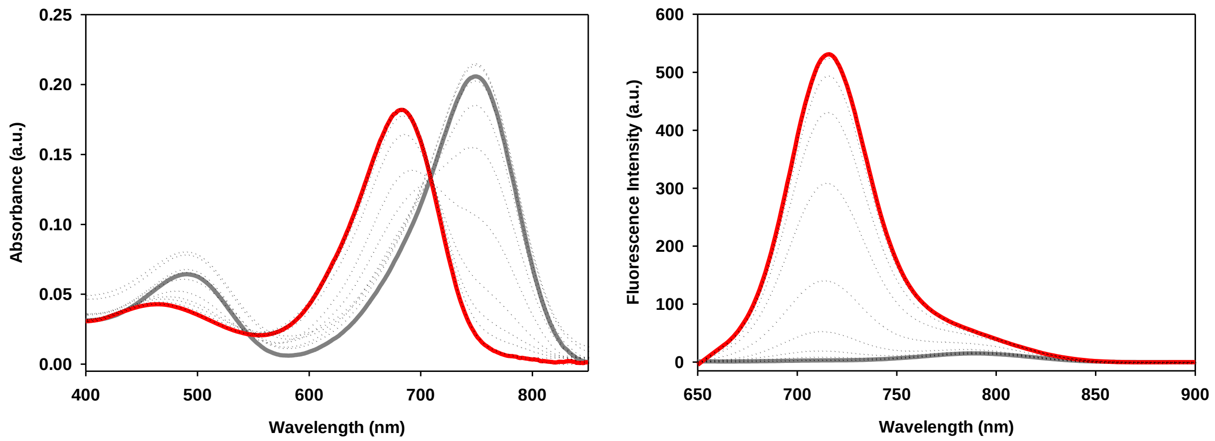

Absorbance (left) and fluorescence (right) spectra of **1c** in PBS buffer/TX-100 (0.34 mM) starting at pH 8 (grey line) to pH 2 (red line). Fluorescence excitation: 630 nm; range: 650 - 900; slit widths: 5/5. Apparent pKa = 4.7.

Figure S4. Widefield microscopy imaging of **1a-c**

Time course of widefield imaging MDA-MB 231 cells treated with **1a** (5 μM)

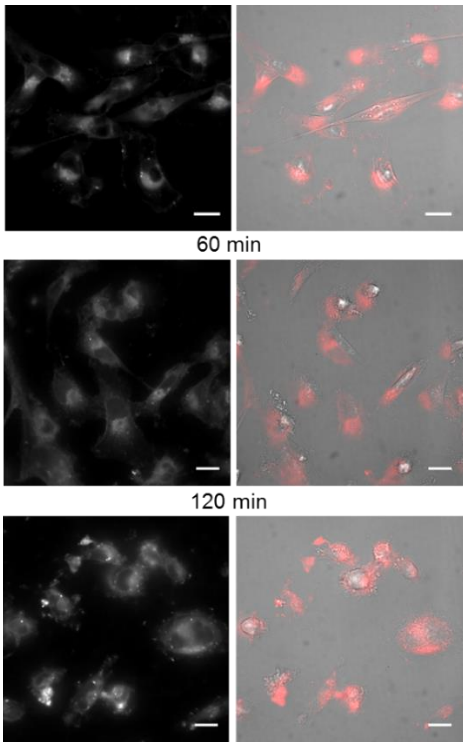

Time course of widefield imaging MDA-MB 231 cells treated with **1b** (5 μM)

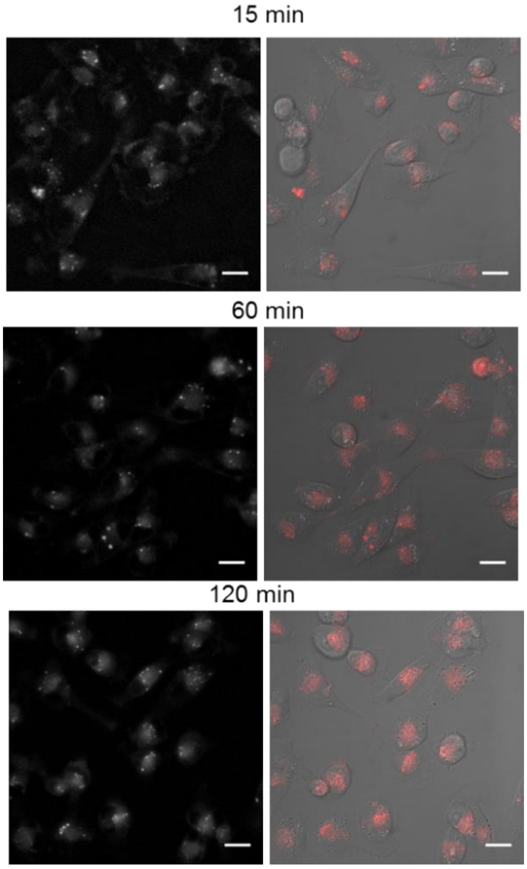

Time course of widefield imaging MDA-MB 231 cells treated with **1c** (5 μM).

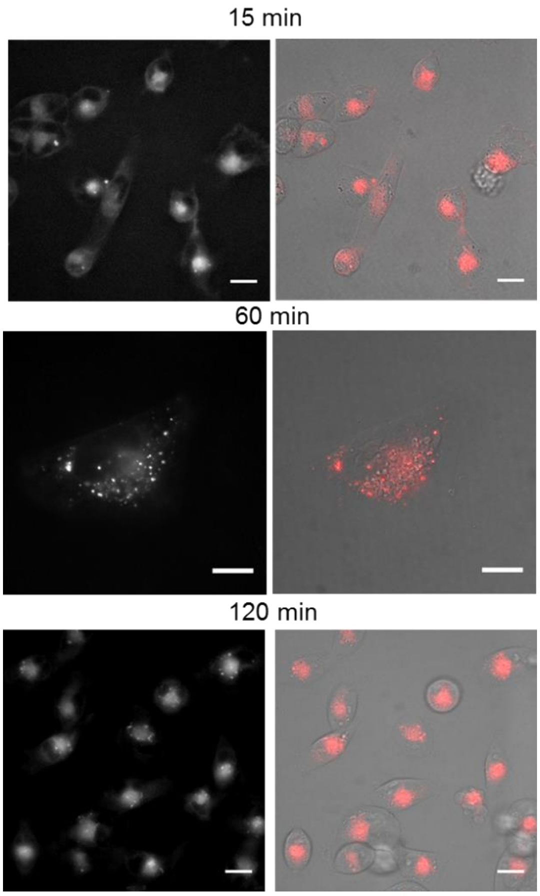

FigureS5. Plots showing measured tumor emission intensities over time for **1a** (green traces), **1b** (red traces) **1c** (blue traces).

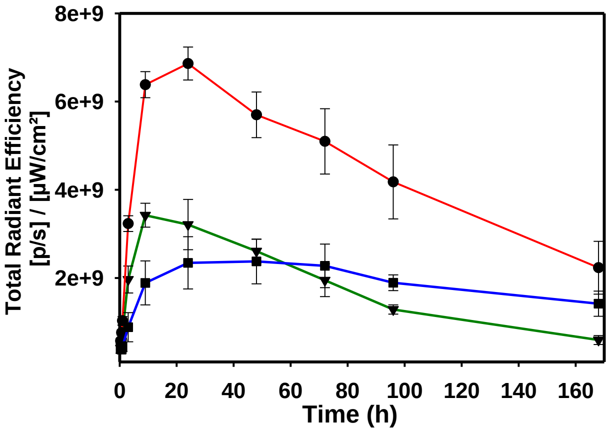

Figure S6. TBR analysis of *in vivo* fluorescence imaging for *always-on* PEG NIR-AZA **2a**. Values determined by ROI total fluorescence signal of tumor divided by an averaged value of three background regions as measured by Living Image Software v4.7.

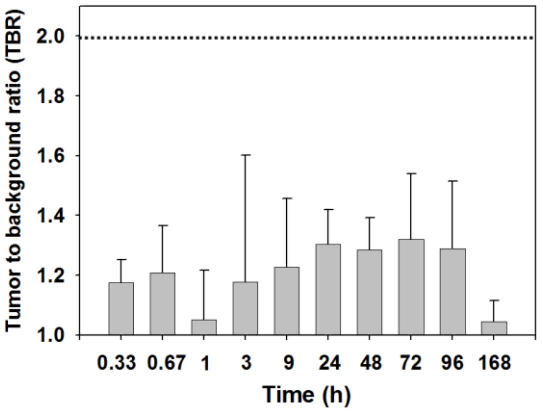

Figure S7. Fluorescence image of excised tumor from animal treated with **1c**, 168 h post administration.

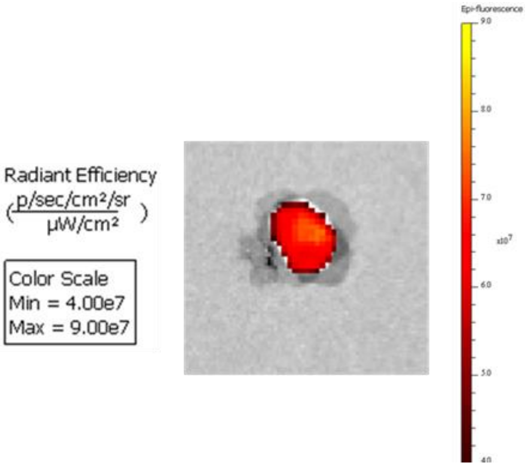

Figure S8. Fluorescence intensity of tumor and excised organs for **1a** (at 9 h post administration) and **1b** (at 24 h post administration) (n=2).

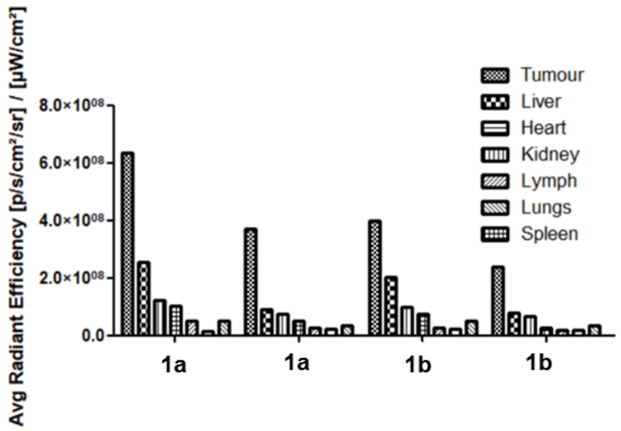

Supplement: Supplementary file 1 — Supplementary figures and movie legends. [file thnov10p3064s1.pdf]
